# Childhood psychosocial adjustment and midlife obesity, diabetes and hypertension: prospective study from two birth cohorts

**DOI:** 10.1192/bjp.2024.133

**Published:** 2024-12

**Authors:** Lin Liu, Kevin Chun Hei Wu, Anping Cai, Aimin Xu, Bernard M. Y. Cheung

**Affiliations:** Department of Medicine, School of Clinical Medicine, The University of Hong Kong, Pokfulam, Hong Kong, China; Hypertension Research Laboratory, Department of Cardiology, Guangdong Cardiovascular Institute, Guangdong Provincial People's Hospital, Guangdong Academy of Medical Sciences, Southern Medical University, Guangzhou, Guangdong Province, China; Department of Medicine, School of Clinical Medicine, The University of Hong Kong, Pokfulam, Hong Kong, China; and State Key Laboratory of Pharmaceutical Biotechnology, The University of Hong Kong, Pokfulam, Hong Kong, China; Department of Medicine, School of Clinical Medicine, The University of Hong Kong, Pokfulam, Hong Kong, China; State Key Laboratory of Pharmaceutical Biotechnology, The University of Hong Kong, Pokfulam, Hong Kong, China; and Institute of Cardiovascular Science and Medicine, The University of Hong Kong, Pokfulam, Hong Kong, China

**Keywords:** Childhood psychosocial adjustment, cardiometabolic diseases, mediating factors, birth cohort, longitudinal study

## Abstract

**Background:**

Understanding how childhood psychosocial adjustment (CPA) influences later life health outcomes is crucial for developing interventions to mitigate the long-term risk of cardiometabolic diseases (CMDs).

**Aims:**

To investigate the association between CPA and incident CMDs in mid-life, and the mediating roles of educational attainment, smoking habits and depression during young adulthood.

**Method:**

A prospective cohort study utilised data from the 1958 National Child Development Study (NCDS; 1958–2013) and the 1970 British Cohort Study (BCS70; 1970–2018), encompassing 22 012 participants assessed for CPA in childhood, who were subsequently evaluated for educational attainment, smoking habits and depression in young adulthood, followed by assessments for CMDs in mid-life. CPA was assessed using the Bristol Social Adjustment Guides in the NCDS and the Rutter Child Behaviour Scale in the BCS70, with higher scores indicating poorer psychosocial adjustment. The primary outcomes were the mid-life incidences of hypertension, diabetes and obesity.

**Results:**

Compared with children in the lowest tertile for CPA scores, those in the middle tertile had an adjusted odds ratio for hypertension of 0.98 (95% CI 0.90–1.06), whereas those in the highest tertile had an odds ratio of 1.17 (95% CI 1.08–1.26). For diabetes, the corresponding odds ratios (95% CI) were 1.15 (0.98–1.35) and 1.39 (1.19–1.62). For obesity, the corresponding odds ratios (95% CI) were 1.08 (1.00–1.16) and 1.18 (1.09–1.27). These associations were partially mediated by educational attainment (2.4–13.9%) and depression during young adulthood (2.5–14.9%).

**Conclusions:**

Poorer CPA is correlated with the development of hypertension, diabetes and obesity in mid-life. Interventions aimed at improving CPA may help in reducing the burden of these diseases in later life.

Cardiometabolic diseases (CMDs) are among the foremost causes of morbidity and mortality globally, placing a significant strain on healthcare systems. Among the CMDs, the hypertension–diabetes continuum is the leading risk factor for atherosclerosis, and obesity has been identified as the most important risk factor for hypertension and diabetes.^1^ The sustained increase in the global public health burden of these conditions highlights the critical need to understand their development, which is essential for implementing effective early intervention strategies. Recent research has shed light on how early-life experiences, such as adverse childhood events, emotional disturbances and attention-deficit hyperactivity disorder (ADHD), act as persistent stressors that can elevate the risk of developing CMDs later in life,^2,3^ thereby underscoring the influence of early-life psychosocial factors in the pathogenesis of CMDs.

Childhood psychosocial adjustment (CPA) encompasses the development of social and emotional competencies that enable children to effectively manage their relationships with peers, family and others in their surroundings.^4^ Typically assessed in familial and educational settings, CPA is a multifaceted construct that includes both internalising behaviours, such as anxiety and depression, and externalising behaviours, such as aggression and hyperactivity.^5^ The development of CPAs is shaped by a complex interplay of genetic predispositions, family dynamics, parenting practices and peer interactions.^6–8^ Poor CPA has been associated with elevated adverse cardiovascular biomarkers and premature mortality.^9^ The concept of biological embedding plays a crucial role in this context, proposing that early childhood stressors and adaptive responses can become ‘embedded’ in the body's physiology, potentially modifying gene expression (epigenetics), altering neural development, and affecting the functionality of immune, metabolic and cardiovascular systems.^10^

The long-term effects of poor CPA on CMDs are profound, suggesting that traits observed in early adulthood could mediate this relationship. For instance, children with ADHD are more likely to start smoking early, achieve lower educational levels and develop depressive disorders in early adulthood – factors known to increase the risk of CMDs.^11,12^

However, there is a notable paucity of large-scale, long-term prospective studies tracing the relationship between CPA and CMDs from birth to mid-life. Utilising data from two UK birth cohorts – the 1958 National Child Development Study (NCDS) and the 1970 British Cohort Study (BCS70) – our research aims to estimate the association between CPA (as reflected in performance in family and school settings) and mid-life CMDs. Additionally, we seek to evaluate the potential mediating roles of educational attainment, smoking habits and depression during young adulthood in this association.

## Method

### Study population

**Fig. 1:**
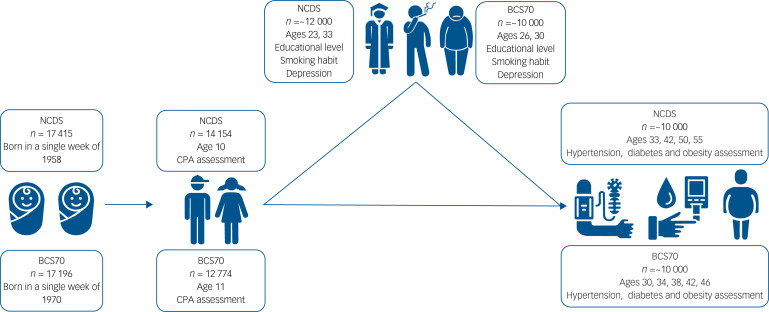
Study flowchart. NCDS, National Childhood Development Study; BCS70, 1970 British Cohort Study; CPA, childhood psychosocial adjustment.

We analysed data from two longitudinal UK birth cohorts: the 1958 National Child Development Study (NCDS) and the 1970 British Cohort Study (BCS70). These ongoing multidisciplinary surveys captured data from 17 415 and 17 196 individuals born in a single week in 1958 and 1970 respectively.^13,14^ The NCDS has collected data across eight waves – at ages 0, 7, 10, 23, 33, 42, 50 and 55 years – and the BCS70 includes nine waves – at ages 0, 5, 11, 26, 30, 34, 38, 42 and 46 years. The study questions concerning all variables by cohort are presented in Supplementary Table 1, available at https://doi.org/10.1192/bjp.2024.133. We set the age 10 wave in the NCDS and the age 11 wave in the BCS70 as the respective baselines. The study flowchart and response rates in both cohorts are depicted in Fig. 1.

This study did not require ethics approval or informed consent from the participants because we used publicly available secondary data. However, approval had been sought from both institutional review and/or internal ethical review boards, and participants’ informed consent had been obtained, for NCDS and BCS70 data collection.

### Exposure

Childhood psychosocial adjustment (CPA) was measured using the Bristol Social Adjustment Guides (BSAG) at age 11 for the NCDS and the Rutter Child Behaviour Scale (RCBS) at age 10 for the BCS70. The BSAG is a comprehensive booklet with approximately 250 behavioural descriptors, where teachers identify relevant behaviours for each child: a CPA score was generated by summing the number of coded items. The RCBS comprises 19 behavioural descriptors, with mothers indicating the extent to which each applies to their child via a visual analogue scale: a CPA score was created by summing the scores of all 19 descriptors. Higher scores on both scales signify worse CPA. CPA was further categorised into four subtypes: conduct problems, emotional problems, hyperactivity and miscellaneous, with higher scores indicating greater severity for each subtype (Supplementary Table 2). Details of the BSAG and RCBS, as well as the definitions for subtypes, are provided in the Supplementary Method.

### Mediators

The mediators linking CPA and CMDs can include behavioural, socioeconomic and psychological factors. To minimise redundancy and overestimation of indirect effects, we selected specific proxies: smoking habits during young adulthood (ever smoking versus never smoking), educational attainment (O level or below versus A level or above) and depressive symptoms in young adulthood (with versus without depression). The choice of smoking habits was informed by its significant public health implications and direct health risks. The proportion of daily cigarette smokers who transitioned to daily smoking in early adulthood has increased from 38.7% in 2002 to 55.9% in 2018 in the USA.^15^ Although sleep patterns, diet and physical inactivity are also critical behavioural indicators, their effects are more diffuse and pose greater challenges in precise measurement compared with smoking. Educational attainment, once achieved, remains relatively stable over time and reflects a long-term investment in skills and knowledge. It serves as a stable and enduring indicator of socioeconomic status, particularly in younger populations. Finally, depression captures a wide range of psychological distress and is generally more debilitating than other psychological factors, such as anxiety or personality disorders, making it a critical proxy for assessing psychological impacts. Mediators were evaluated at ages 23 and 33 in the NCDS and at ages 26 and 30 in the BCS70. Details on the questions and definitions for these mediators are given in the Supplementary Method.

### Outcomes

Our study focused on three cardiometabolic diseases: hypertension, diabetes and obesity. The selection of these outcomes was driven by their significant public health impact and high prevalence among young adults, unlike other severe CMDs such as stroke and myocardial infarction, which predominantly affect older adults. Additionally, these conditions were chosen because of their potential associations with the exposures and mediators being investigated in our study. In the NCDS, hypertension and diabetes were self-reported at ages 33, 42, 50 and 55, and obesity was defined as a body mass index BMI ≥ 30 kg/m², calculated from self-reported weight and height at these ages (with height at age 42 used for subsequent waves owing to the lack of assessment). For the BCS70, these conditions were self-reported at ages 30, 34, 42 and 46, and obesity was similarly defined based on BMI at these ages. Participants meeting the criteria at any wave were classified accordingly.

### Potential confounders

Potential confounders were chosen for their potential to confound the relationship between CPA and CMDs. These include demographic factors such as gender and region of residence, socioeconomic factors such as parental social class, prenatal factors such as maternal smoking during pregnancy, method of delivery, birth weight and breastfeeding history. Additionally, exposure to tobacco smoke during childhood was considered. The selection of these factors was guided by previous research that have identified them as significant confounders in the link between early psychosocial factors and mid-life health outcomes,^9^ as well as the data availability in our study. These variables, except for breastfeeding history and childhood exposure to tobacco smoke, were derived from initial waves of both cohorts, with details provided in the Supplementary Method.

### Statistical analysis

Participants were grouped into tertiles based on their total CPA scores within each cohort. Characteristics are presented as means or medians for continuous variables and frequencies for categorical variables.

We conducted logistic regression to explore the association between CPA scores and mid-life CMDs, using the lowest tertile as the reference. Two models were applied: model 1 without adjustments and model 2 adjusted for gender, region of residence, social class, maternal smoking during pregnancy, method of delivery, birth weight and breastfeeding (with childhood exposure to tobacco smoke further adjusted in BCS70 models). Missing covariate data were addressed through multiple imputation (*m* = 5), with odds ratios and 95% confidence intervals pooled from all imputed data-sets. Regressions were performed separately for each cohort, followed by a fixed-effects meta-analysis of individual participant data.

Causal mediation analysis was employed to assess the mediating effects of our three mediators on the CPA–mid-life CMDs relationship among participants with complete data on exposure, mediators and outcomes. Separate analyses were conducted for each cohort, treating CPA score tertiles as continuous (valued at 1, 2 and 3), with gender and region of residence as confounders. The proportions of mediating effects and 95% confidence intervals were reported.

For the relationship between CPA and mid-life CMDs, sensitivity analyses were performed in two ways. First, fully adjusted models were fit by gender (male and female) and parental social class (I/II, IIIa/b and IV/V) within each cohort, followed by meta-analysis. Second, fully adjusted models were fit after multiple imputation (*m* = 20) for outcomes and aggregated via meta-analysis. For mediation analysis, we conducted sensitivity analyses by treating CPA tertiles as binary variables: setting the first tertile as one dummy variable and combining the first and second tertiles as another dummy variable for separate mediation analyses.

A two side *P*-value of <0.05 was considered statistically significant. All analyses were conducted using R 4.3.1 for Windows.

## Results

### Participant characteristics

Our study comprised 14 154 individuals from the National Child Development Study (NCDS) and 12 774 from the British Cohort Study 1970 (BCS70), with a balanced gender distribution (46.8% girls in both cohorts). Supplementary Table 3 provides a summary of participant characteristics, including early-life factors, mediators in young adulthood and mid-life outcomes across tertiles of childhood psychosocial adjustment (CPA) scores. Notably, there was approximately a 20% rate of missing data for mediators and outcomes. Baseline characteristics according to the presence of hypertension, diabetes and obesity by cohort are detailed in Supplementary Tables 4–9. Higher CPA scores in childhood were associated with the development of these conditions in mid-life for both cohorts.

### CPA and midlife cardiometabolic diseases (CMDs)

Table 1 illustrates the relationship between CPA and mid-life CMDs. In the NCDS, higher CPA scores were linked to an increased likelihood of hypertension, diabetes and obesity after adjusting for confounding factors. In the BCS70, a similar increase was found for hypertension. Meta-analyses of NCDS and BCS70 cohorts revealed significant associations. For hypertension, compared with those in the lowest tertile of CPA scores, the odds ratio for the middle tertile was 0.98 (95% CI 0.90–1.06), and for the highest tertile it was 1.17 (95% CI 1.08–1.26). For diabetes, the corresponding odds ratios were 1.15 (95% CI 0.98–1.35) and 1.39 (95% CI 1.19–1.62), and for obesity they were 1.08 (95% CI 1.00–1.16) and 1.18 (95% CI 1.09–1.27).

**Table 1:** Association between childhood psychosocial adjustment (CPA) and mid-life cardiometabolic diseases for the National Childhood Development Study (NCDS) and 1970 British Cohort Study (BCS70) meta-analyses^a^

National Childhood Development Study						
	Hypertension (*n* = 11 427)		Diabetes (*n* = 11 427)		Obesity (*n* = 11 006)	
CPA score	Model 1^b^	Model 2^c^	Model 1^b^	Model 2^c^	Model 1^b^	Model 2^c^
Tertile 1	Ref	Ref	Ref	Ref	Ref	Ref
Tertile 2	0.93 (0.84–1.03)	0.92 (0.83–1.02)	1.12 (0.91–1.37)	1.09 (0.88–1.34)	1.13 (1.03–1.25)	1.10 (1.00–1.22)
Tertile 3	1.14 (1.03–1.26)	1.11 (1.00–1.23)	1.60 (1.32–1.94)	1.49 (1.22–1.81)	1.33 (1.20–1.47)	1.26 (1.14–1.39)
*P*-trend	0.01	0.05	<0.001	<0.001	<0.001	<0.001

1970 British Cohort Study						
	Hypertension (*n* = 11 427)		Diabetes (*n* = 11 427)		Obesity (*n* = 11 006)	
CPA score	Model 1^b^	Model 2^d^	Model 1^b^	Model 2^d^	Model 1^b^	Model 2^d^
Tertile 1	Ref	Ref	Ref	Ref	Ref	Ref
Tertile 2	1.08 (0.95–1.23)	1.07 (0.94–1.22)	1.27 (0.98–1.63)	1.24 (0.97–1.60)	1.08 (0.97–1.20)	1.06 (0.95–1.18)
Tertile 3	1.31 (1.16–1.49)	1.26 (1.11–1.43)	1.33 (1.04–1.70)	1.24 (0.97–1.60)	1.15 (1.03–1.28)	1.08 (0.97–1.21)
*P*-trend	<0.001	<0.001	0.02	0.09	0.005	0.16

National Childhood Development Study and 1970 British Cohort Study meta-analyses						
	Hypertension (*n* = 22 012)		Diabetes (*n* = 22 012)		Obesity (*n* = 21 196)	
CPA score	Model 1^e^	Model 2^f^	Model 1^e^	Model 2^f^	Model 1^e^	Model 2^f^
Tertile 1	Ref	Ref	Ref	Ref	Ref	Ref
Tertile 2	0.99 (0.91–1.07)	0.98 (0.90–1.06)	1.19 (1.01–1.39)	1.15 (0.98–1.35)	1.08 (1.01–1.17)	1.08 (1.00–1.16)
Tertile 3	1.17 (1.08–1.26)	1.17 (1.08–1.26)	1.44 (1.24–1.68)	1.39 (1.19–1.62)	1.20 (1.12–1.29)	1.18 (1.09–1.27)

Ref, reference.

a. Results are presented as odds ratios with 95% confidence intervals in parentheses.

b. Unadjusted models.

c. Models were adjusted for gender, region of residence, social class, maternal smoking during pregnancy, method of delivery, birth weight and breastfeeding.

d. Models were adjusted for gender, region of residence, social class, maternal smoking during pregnancy, method of delivery, birth weight, breastfeeding and childhood exposure to tobacco smoke.

e. Log-ORs and variance were derived from unadjusted logistic models in NCDS and BCS70 respectively.

f. Log-ORs and variance were derived from adjusted logistic models in NCDS and BCS70 respectively.

Fixed effects were considered across studies. All *P*-values for heterogeneity >0.1.

### CPA subtypes and midlife CMDs

Table 2 presents the associations between four CPA subtypes and mid-life CMDs, showing variable precision depending on the subtype, CMD and cohort. Meta-analyses for the NCDS and BCS70 cohorts indicated that conduct problems were associated with increased odds of hypertension and diabetes, with the highest tertile compared with the lowest showing odds ratios of 1.08 (95% CI 1.00–1.17) and 1.19 (95% CI 1.02–1.39) respectively. Emotional problems were linked to all three conditions, with odds ratios of 1.16 (95% CI 1.07–1.25) for hypertension, 1.33 (95% CI 1.15–1.55) for diabetes and 1.17 (95% CI 1.09–1.26) for obesity. Hyperactivity and miscellaneous problems were similarly associated with increased odds for these CMDs.

**Table 2:** Association between subtypes of childhood psychosocial adjustment (CPA) and mid-life cardiometabolic diseases for the National Childhood Development Study (NCDS) and 1970 British Cohort Study (BCS70) meta-analyses^a^

	Hypertension					
	NCDS		BCS70		NCDS and BSC70 meta-analyses	
CPA type	Model 1^b^	Model 2^c^	Model 1^b^	Model 2^d^	Model 1^e^	Model 2^f^
Conduct problems						
Tertile 1	Ref	Ref	Ref	Ref	Ref	Ref
Tertile 2	1.08 (0.98–1.20)	1.07 (0.97–1.19)	1.04 (0.91–1.18)	1.02 (0.90–1.16)	1.07 (0.98–1.15)	1.05 (0.97–1.14)
Tertile 3	1.10 (0.99–1.22)	1.08 (0.97–1.19)	1.14 (1.00–1.29)	1.08 (0.95–1.23)	1.12 (1.03–1.21)	1.08 (1.00–1.17)
Emotional problems						
Tertile 1	Ref	Ref	Ref	Ref	Ref	Ref
Tertile 2	1.01 (0.91–1.12)	1.01 (0.91–1.12)	1.06 (0.93–1.21)	1.06 (0.93–1.20)	1.03 (0.95–1.12)	1.03 (0.95–1.11)
Tertile 3	1.17 (1.05–1.29)	1.14 (1.03–1.27)	1.21 (1.07–1.37)	1.18 (1.04–1.34)	1.18 (1.09–1.28)	1.16 (1.07–1.25)
Hyperactivity						
Tertile 1	Ref	Ref	Ref	Ref	Ref	Ref
Tertile 2	1.04 (0.94–1.16)	1.04 (0.94–1.16)	1.03 (0.91–1.18)	1.03 (0.90–1.17)	1.04 (0.96–1.13)	1.04 (0.96–1.13)
Tertile 3	1.06 (0.96–1.18)	1.04 (0.94–1.16)	1.27 (1.12–1.43)	1.22 (1.07–1.39)	1.14 (1.05–1.23)	1.11 (1.02–1.20)
Miscellaneous						
Tertile 1	Ref	Ref	Ref	Ref	Ref	Ref
Tertile 2	0.97 (0.87–1.08)	0.97 (0.88–1.08)	1.09 (0.96–1.24)	1.09 (0.96–1.24)	1.02 (0.94–1.10)	1.02 (0.94–1.10)
Tertile 3	1.11 (1.01–1.23)	1.10 (1.00–1.22)	1.19 (1.05–1.35)	1.15 (1.01–1.31)	1.14 (1.05–1.24)	1.12 (1.03–1.21)

	Diabetes					
	NCDS		BCS70		NCDS and BSC70 meta-analyses	
	Model 1^b^	Model 2^c^	Model 1^b^	Model 2^d^	Model 1^e^	Model 2^f^
Conduct problems						
Tertile 1	Ref	Ref	Ref	Ref	Ref	Ref
Tertile 2	1.10 (0.90–1.34)	1.06 (0.87–1.30)	1.14 (0.89–1.46)	1.12 (0.88–1.44)	1.12 (0.96–1.30)	1.09 (0.93–1.27)
Tertile 3	1.33 (1.10–1.61)	1.25 (1.03–1.52)	1.18 (0.92–1.51)	1.09 (0.85–1.40)	1.27 (1.09–1.48)	1.19 (1.02–1.39)
Emotional problems						
Tertile 1	Ref	Ref	Ref	Ref	Ref	Ref
Tertile 2	1.22 (1.00–1.50)	1.20 (0.98–1.48)	0.94 (0.73–1.21)	0.94 (0.73–1.21)	1.10 (0.94–1.29)	1.09 (0.93–1.28)
Tertile 3	1.56 (1.28–1.90)	1.47 (1.21–1.79)	1.19 (0.94–1.51)	1.16 (0.91–1.47)	1.40 (1.2–1.63)	1.33 (1.15–1.55)
Hyperactivity						
Tertile 1	Ref	Ref	Ref	Ref	Ref	Ref
Tertile 2	0.99 (0.81–1.21)	0.98 (0.80–1.20)	0.94 (0.73–1.21)	0.94 (0.73–1.21)	0.97 (0.83–1.14)	0.96 (0.82–1.13)
Tertile 3	1.30 (1.07–1.57)	1.20 (1.00–1.46)	1.22 (0.96–1.55)	1.13 (0.89–1.44)	1.27 (1.09–1.47)	1.17 (1.01–1.37)
Miscellaneous						
Tertile 1	Ref	Ref	Ref	Ref	Ref	Ref
Tertile 2	0.85 (0.69–1.04)	0.86 (0.70–1.05)	1.27 (0.99–1.64)	1.28 (0.99–1.64)	1.00 (0.85–1.17)	1.00 (0.86–1.18)
Tertile 3	1.39 (1.16–1.68)	1.36 (1.13–1.64)	1.42 (1.11–1.83)	1.41 (1.10–1.81)	1.41 (1.21–1.63)	1.38 (1.19–1.60)

	Obesity					
	NCDS		BCS70		NCDS and BSC70 Meta-analyses	
	Model 1^b^	Model 2^c^	Model 1^b^	Model 2^d^	Model 1^e^	Model 2^f^
Conduct problems						
Tertile 1	Ref	Ref	Ref	Ref	Ref	Ref
Tertile 2	1.07 (0.97–1.18)	1.03 (0.94–1.14)	1.04 (0.93–1.15)	1.01 (0.91–1.13)	1.05 (0.98–1.13)	1.03 (0.95–1.10)
Tertile 3	1.06 (0.96–1.17)	1.01 (0.91–1.12)	1.15 (1.03–1.28)	1.07 (0.96–1.20)	1.10 (1.03–1.19)	1.04 (0.96–1.12)
Emotional problems						
Tertile 1	Ref	Ref	Ref	Ref	Ref	Ref
Tertile 2	1.03 (0.94–1.14)	1.02 (0.93–1.13)	0.97 (0.87–1.08)	0.96 (0.86–1.07)	1.00 (0.93–1.08)	0.99 (0.92–1.07)
Tertile 3	1.40 (1.27–1.54)	1.34 (1.22–1.48)	1.01 (0.91–1.13)	0.99 (0.89–1.10)	1.21 (1.12–1.30)	1.17 (1.09–1.26)
Hyperactivity						
Tertile 1	Ref	Ref	Ref	Ref	Ref	Ref
Tertile 2	1.12 (1.02–1.24)	1.12 (1.01–1.23)	0.95 (0.85–1.06)	0.94 (0.85–1.05)	1.04 (0.97–1.12)	1.03 (0.96–1.11)
Tertile 3	1.39 (1.26–1.54)	1.34 (1.21–1.48)	1.17 (1.06–1.31)	1.12 (1.00–1.24)	1.29 (1.20–1.38)	1.23 (1.14–1.32)
Miscellaneous						
Tertile 1	Ref	Ref	Ref	Ref	Ref	Ref
Tertile 2	0.99 (0.9–1.09)	1.00 (0.90–1.10)	0.99 (0.89–1.10)	0.98 (0.88–1.10)	0.99 (0.92–1.06)	0.99 (0.92–1.07)
Tertile 3	1.17 (1.06–1.29)	1.14 (1.04–1.26)	1.10 (0.98–1.22)	1.07 (0.96–1.20)	1.13 (1.06–1.22)	1.11 (1.03–1.20)

Ref, reference.

a. Results are presented as odds ratios (ORs) with 95% confidence intervals in parentheses.

b. Unadjusted models.

c. Models were adjusted for gender, region of residence, social class, maternal smoking during pregnancy, method of delivery, birth weight and breastfeeding.

d. Models were adjusted for gender, region of residence, social class, maternal smoking during pregnancy, method of delivery, birth weight, breastfeeding and childhood exposure to tobacco smoke.

e. Log-ORs and variance were derived from unadjusted logistic models in NCDS and BCS70 respectively.

f. Log-ORs and variance were derived from adjusted logistic models in NCDS and BCS70 respectively.

Higher scores indicate more severe problems. Fixed effects were considered across studies. All *P*-values for heterogeneity >0.1.

### Mediation analysis

Table 3 shows the mediation analysis results by cohort. In the NCDS, low educational levels accounted for a small but significant percentage of the association between CPA and CMDs: 2.4% for hypertension, 6.7% for diabetes and 13.9% for obesity. Depression also served as a mediator, particularly for the association between CPA and diabetes, explaining 7.3% of the association. In the BCS70, low educational levels explained 1.9% of the association with hypertension and 8.0% with obesity. Depression was a more substantial mediator for hypertension (10.9%), diabetes (14.5%) and obesity (10.0%). Smoking habits did not significantly mediate the relationship between CPA and the CMDs in question for either cohort.

**Table 3:** Mediation analysis according to cohorts

Outcome	Mediators	Mediation proportion (95% CI)	
		NCDS	BCS70
Hypertension	Smoking habit^a^	1.0 (−6.1 to 11.0)	0.5 (−0.6 to 3.0)
	Educational levels^b^	2.4 (0.9 to 11.1)*	1.9 (0.1 to 6.0)*
	Depression^c^	2.5 (0.6 to 14.5)*	10.9 (5.1 to 24.0)**
Diabetes	Smoking habit^a^	0.1 (−4.2 to 4.0)	−0.1 (−15.8 to 13.0)
	Educational levels^b^	6.7 (1.2 to 16.0)*	−0.3 (−6.5 to 6.0)
	Depression^c^	7.3 (3.1 to 18.0)**	14.5 (4.1 to 79.0)*
Obesity	Smoking habit^a^	−0.7 (−4.3 to 3.0)	−1.5 (−8.1 to 1.0)
	Educational levels^b^	13.9 (8.2 to 24.0)**	8.0 (1.4 to 37.0)*
	Depression^c^	2.1 (−0.2 to 5.0)	10.0 (3.1 to 43.0)**

NCDS, National Childhood Development Study; BCS70, 1970 British Cohort Study.

a. Ever smoking versus never smoking.

b. Highest education O level or below versus A level or above.

c. Depression versus not depression.

Gender and residential region were adjusted as confounders.

*P < 0.05, **P < 0.01.

### Sensitivity analysis

Sensitivity analyses for logistic regression indicate that the positive associations between CPA scores and odds of mid-life CMDs remained stable regardless of gender (Supplementary Tables 10–12) and social class (Supplementary Tables 13–15) and in analyses using fully imputed data (Supplementary Table 16). Mediation analysis results were also robust in sensitivity analysis (Supplementary Tables 17 and 18).

## Discussion

### Principal findings

Our study provides a thorough analysis of how childhood psychosocial adjustment (CPA) affects the likelihood of developing cardiometabolic diseases (CMDs) during mid-life. Utilising data from two sizeable birth cohorts, our findings indicate a significant association between poor CPA and an elevated likelihood of hypertension, diabetes and obesity in mid-life. Specifically, emotional problems, hyperactivity and miscellaneous CPA subtypes were linked to all three CMDs, while conduct problems correlated with hypertension and diabetes. Additionally, mediation analysis suggested that both lower educational attainment and early-life depression partially contributed to the connection between CPA and CMDs

### Comparison with other studies

In support of our results, prior research has identified various CPA factors (e.g. conduct problems, mental health problems, attention-deficit hyperactivity disorder (ADHD)) as predictors of adverse adult outcomes.^12,16,17^ Colman and colleagues noted that adolescents with severe externalising behaviours face a range of social and health challenges, including mental health issues, strained family life, and poor employment and educational outcomes in adulthood,^18^ which also extend to physical health.^19^ Similarly, Hoffmann and colleagues found enduring links between adolescent mental health problems (reported by parents or teachers) and negative educational, economic, social, health and psychological outcomes later in life.^20^ Recent meta-analyses have also connected ADHD with adverse academic, mental health, substance use and employment outcomes.^21^ Nevertheless, the evidence directly associating CPA with mid-life CMDs remains scarce. Drawing on the National Child Development Study (NCDS), Ploubidis and colleagues explored early-life mental health's relationship with cardiometabolic biomarkers at age 42, identifying associations with elevated fibrinogen levels and blood pressure.^9^ Thapar and colleagues also reported correlations between ADHD symptoms and higher body mass index (BMI), blood pressure and triglyceride levels, using the same analytical approach as Ploubidis et al.^22^ Our study extends and refines this body of evidence, delivering a detailed evaluation of childhood psychosocial conditions, integrating data from all life-course stages, rather than a single time point, to capture comprehensive information on hypertension, diabetes and obesity. Moreover, besides using NCDS data, we explored this relationship with the 1970 British Cohort Study (BCS70) and conducted a meta-analysis of these cohorts to further strengthen the reliability and validity of our findings.

### Potential mechanism

The mechanisms through which poor CPA might be associated with increased odds of CMDs are likely multifaceted. Our analysis indicates that lower educational attainment partly mediates this relationship. Education, a precursor to socioeconomic factors such as occupation and income, is crucial for maintaining healthy lifestyles and accessing healthcare.^23^ Individuals with lower educational attainment may be more likely to occupy jobs that are physically demanding, low-paid and offer little control, which are stressors that have been linked to poor health outcomes.^24^ Limited income also restricts access to healthcare, nutritious food and safe living environments, further heightening the risk of CMDs.^25^ Furthermore, individuals with lower educational levels are more likely to smoke, have poor diets and lead sedentary lifestyles, and they are also less likely to follow medical advice,^26^ which diminishes the effectiveness of CMD prevention measures in primary and secondary care, leaving them more susceptible to these diseases. Early adulthood depression also emerged as a notable mediator in the CPA–CMD relationship. Depression can encourage development of CMDs through behavioural pathways (e.g. physical inactivity, unhealthy eating)^27,28^ and has significant biological impacts on the development of CMDs. For instance, depression is associated with increased systemic inflammation, insulin resistance and endothelial dysfunction, all of which contribute to CMDs.^29^ Our findings did not identify early-life smoking as a significant mediator in the CPA–CMD relationship, which contrasts with previous studies that have highlighted smoking as a critical risk factor for CMDs. This discrepancy might arise from two factors. First, our study defined smoking habits as either ‘ever smoking’ or ‘never smoking’, which may not account for those who quit smoking later, thus reducing their duration of exposure. Second, our participants were relatively young (aged ≤55 years in the NCDS and ≤46 in BCS70), meaning the cumulative harm from smoking was likely lower and its dose-response effect on additional cardiometabolic risks had not fully manifested.^30^ It is important to acknowledge that other factors, including sleep patterns, diet and physical inactivity, may also serve as mediators. Further research is essential to explore and quantify these additional indirect pathways connecting CPA to CMDs.

Apart from the indirect effect between global CPA and mid-life CMDs, our study also suggested direct effects linking subtypes of CPA to mid-life CMDs. Specifically, childhood hyperactivity significantly increased the odds of hypertension, diabetes and obesity in mid-life. Although hyperactivity might intuitively seem protective against obesity owing to increased physical activity, observational studies have shown that those with ADHD often experience dysregulation of eating behaviour.^31^ Additionally, recent studies indicate genetic overlaps between ADHD and BMI,^32^ and a causal relationship between childhood ADHD and obesity was revealed by a Mendelian randomisation study.^3^ Childhood hyperactivity also increased the odds of hypertension. Besides the similar pathways shared with obesity, medications used to treat hyperactivity may partly account for this relationship.^33^ However, some studies have found conflicting results, suggesting that hyperactivity could be protective against obesity and showing no significant relationship with blood pressure.^34,35^ These discrepancies indicate that the relationship between hyperactivity and CMDs may be influenced by the severity of exposure and the treatment received. Psychological factors are well-documented risk factors for CMDs. Our findings indicate that children with emotional problems are more likely to develop hypertension, diabetes and obesity in mid-life. Psychological disorders serve as a source of chronic stress, which can alter neuroendocrinological mechanisms such as the hypothalamic–pituitary–adrenal (HPA) axis and the sympathetic nervous system.^36^ Persistent chronic stress can lead to continuous activation of these systems, negatively affecting cardiometabolic functions such as immune response, blood pressure and lipid metabolism.^37^ Long-term epigenetic alterations induced by chronic stress, affecting the HPA system and resulting in elevated cortisol levels, have also been observed.^33,38^ However, a multi-ethnic cross-sectional study found no relationship between depression and diabetes,^39^ suggesting that the effect of emotional problems on CMDs could be dose-responsive, with those experiencing childhood emotional problems being at higher risk of CMDs because of prolonged exposure to chronic stress. Childhood conduct problems also increased the odds of hypertension and diabetes. This may be attributed to the antisocial behaviour patterns developed in childhood that tend to persist across the lifespan, which are associated with poorer health and inadequate healthcare provision.^40^ Our findings of links with all four subtypes suggest that poor CPA could serve as a broad indicator of underlying socioeconomic disadvantages (e.g. poverty, poor living environment) and exposure to chronic stress (e.g. childhood maltreatment, recurrent illness). This broader context aligns with the concept of biological embedding, where early psychosocial stressors influence long-term health outcomes through complex physiological and behavioural pathways.

### Policy implications

The prevention of CMDs presents a multifaceted challenge that necessitates collaboration across various disciplines, such as psychology, paediatrics, sociology, cardiology and endocrinology. Detailed documentation of children's psychosocial health in medical records should be prioritised. These records should be readily accessible to primary healthcare providers, regardless of the patient's age or geographical location. This integration would enable early identification and continuous monitoring of at-risk individuals throughout their lifespan. Children exhibiting psychosocial maladjustment should be identified as a high-risk group for future CMDs, similar to the recognition afforded to smokers and individuals with obesity. This categorisation can inform targeted prevention strategies, including regular screening for CMDs and tailored health interventions. Implementing early intervention programmes that focus on improving CPA is crucial. These programmes should address educational inequalities and mental health issues among young people. Interventions could include school-based mental health services, behavioural therapy and family support programmes. Policymakers should prioritise funding for research and interventions aimed at improving CPA. Investments in mental health services for children and adolescents, particularly in underserved communities, can have long-term benefits in reducing the prevalence of CMDs.

### Strengthens and limitations

A principal strength of our study is the employment of two large, well-characterised cohorts, which bolsters the reliability of our findings. The longitudinal design facilitates a robust temporal analysis of the relationship between CPA and CMDs. Additionally, the extensive data collection process allows for the assessment of a wide range of confounders and mediators. A further notable strength is the intricate classification of CPA subtypes, as reported by parents and teachers, affording a nuanced understanding of how various psychosocial adjustment issues may influence risk of CMDs.

Despite these strengths, several limitations warrant attention. First, the presence of missing data for mediators and outcomes, which amounts to approximately 20%, poses a risk of bias if the missingness correlates with both exposure and outcome. We addressed this issue by employing multiple imputation to create complete data-sets, and our findings remained consistent after pooling the results. Second, there is a persistent possibility of residual confounding despite adjusting for a range of early-life factors in our models. For instance, disparities in social class may have widened during the long-term follow-up. Sensitivity analyses across different social classes confirmed the robustness of the association between CPA and mid-life CMDs, reinforcing our results. Third, although our analysis accounted for select mediators, it did not encompass all conceivable factors, such as diet, physical activity and genetics. We chose three representative mediators for this study: smoking habits (a behavioural factor), educational level (a socioeconomic factor) and depression (a psychological factor). We conducted subgroup analyses to test interaction effects between mediators (Supplementary Table 19) and no interaction effect was found. It is important to acknowledge that the interplay among mediators is complex, and incorporating an excessive number of mediators could lead to redundancy and overestimation of indirect effects. Fourth, the reliance on self-reported measures for certain variables could be perceived as a limitation. To mitigate recall bias, we ascertained early-life factors from the initial survey conducted at birth and assessed CMDs at short intervals. Fifth, caution should be exercised when extrapolating these results to populations outside the UK, since our study focused on two cohorts from the UK. Differences in healthcare systems, socioeconomic factors and lifestyle between countries could significantly affect the generalisability of the findings. Sixth, the use of different tools (the BSAG and RCBS) and different timing to assess childhood psychosocial adjustment across cohorts could lead to measurement inconsistencies. Additionally, the assessment at different ages (10 years old for the NCDS and 11 years old for the BCS70) might introduce a developmental bias. These factors could potentially influence the comparability of the results between cohorts. Last, although our study's prospective design is a strong point, it is essential to recognise that it does not establish causality owing to its observational nature and the potential for residual confounders.

### Conclusion

Our study offers important insights into the relationship between childhood psychosocial adjustment and the odds of developing cardiometabolic diseases (CMDs) in mid-life. The findings suggest that interventions targeting psychosocial health in childhood could have long-term benefits in preventing CMDs.

## Supporting information

Liu et al. supplementary material Liu et al. supplementary material

## Data Availability

The data that support the findings of this study are available from the UK Data Service (https://ukdataservice.ac.uk/).

## References

[1] Cheung BM. The hypertension-diabetes continuum. J Cardiovasc Pharmacol 2010; 55: 333–9. 20422737 10.1097/fjc.0b013e3181d26430

[2] Hughes K, Bellis MA, Hardcastle KA, Sethi D, Butchart A, Mikton C, et al. The effect of multiple adverse childhood experiences on health: a systematic review and meta-analysis. Lancet Public Health 2017; 2: e356–66. 29253477 10.1016/S2468-2667(17)30118-4

[3] Leppert B, Riglin L, Wootton RE, Dardani C, Thapar A, Staley JR, et al. The effect of attention deficit/hyperactivity disorder on physical health outcomes: a 2-sample Mendelian randomization study. Am J Epidemiol 2021; 190: 1047–55. 33324987 10.1093/aje/kwaa273 PMC8168225

[4] Piqueras JA, Mateu-Martínez O, Cejudo J, Pérez-González JC. Pathways into psychosocial adjustment in children: modeling the effects of trait emotional intelligence, social-emotional problems, and gender. Front Psychol 2019; 10: 507. 30915003 10.3389/fpsyg.2019.00507 PMC6423078

[5] Babicka-Wirkus A, Kozlowski P, Wirkus L, Stasiak K. Internalizing and externalizing disorder levels among adolescents: data from Poland. Int J Environ Res Public Health 2023; 20(3): 2752. 36768117 10.3390/ijerph20032752 PMC9915207

[6] Hovey D, Lindstedt M, Zettergren A, Jonsson L, Johansson A, Melke J, et al. Antisocial behavior and polymorphisms in the oxytocin receptor gene: findings in two independent samples. Mol Psychiatry 2016; 21: 983–8. 26390829 10.1038/mp.2015.144

[7] Fonagy P, Butler S, Cottrell D, Scott S, Pilling S, Eisler I, et al. Multisystemic therapy versus management as usual in the treatment of adolescent antisocial behaviour (START): a pragmatic, randomised controlled, superiority trial. Lancet Psychiatry 2018; 5: 119–33. 29307527 10.1016/S2215-0366(18)30001-4 PMC6697182

[8] Etkin RG, Bowker JC. Bidirectional associations between friend overprotection and psychosocial adjustment during adolescence. J Youth Adolesc 2023; 52: 780–93. 36786907 10.1007/s10964-023-01741-6

[9] Ploubidis GB, Batty GD, Patalay P, Bann D, Goodman A. Association of early-life mental health with biomarkers in midlife and premature mortality: evidence from the 1958 British birth cohort. JAMA Psychiatry 2021; 78: 38–46. 32997099 10.1001/jamapsychiatry.2020.2893 PMC7527946

[10] Traniello IM, Robinson GE. Neural and molecular mechanisms of biological embedding of social interactions. Annu Rev Neurosci 2021; 44: 109–28. 34236891 10.1146/annurev-neuro-092820-012959

[11] Rhodes JD, Pelham WE, Gnagy EM, Shiffman S, Derefinko KJ, Molina BS. Cigarette smoking and ADHD: an examination of prognostically relevant smoking behaviors among adolescents and young adults. Psychol Addict Behav 2016; 30: 588–600. 27824233 10.1037/adb0000188 PMC5117481

[12] Galéra C, Bouvard MP, Lagarde E, Michel G, Touchette E, Fombonne E, et al. Childhood attention problems and socioeconomic status in adulthood: 18-year follow-up. Br J Psychiatry 2012; 201: 20–5. 22626635 10.1192/bjp.bp.111.102491 PMC3907305

[13] Elliott J, Shepherd P. Cohort profile: 1970 British birth cohort (BCS70). Int J Epidemiol 2006; 35: 836–43. 16931528 10.1093/ije/dyl174

[14] Power C, Elliott J. Cohort profile: 1958 British birth cohort (national child development study). Int J Epidemiol 2006; 35: 34–41. 16155052 10.1093/ije/dyi183

[15] Barrington-Trimis JL, Braymiller JL, Unger JB, McConnell R, Stokes A, Leventhal AM, et al. Trends in the age of cigarette smoking initiation among young adults in the US from 2002 to 2018. JAMA Netw Open 2020; 3(10): e2019022. 33021650 10.1001/jamanetworkopen.2020.19022 PMC7539122

[16] Clayborne ZM, Varin M, Colman I. Systematic review and meta-analysis: adolescent depression and long-term psychosocial outcomes. J Am Acad Child Adolesc Psychiatry 2019; 58: 72–9. 30577941 10.1016/j.jaac.2018.07.896

[17] Canino GJ, Shrout PE, Wall M, Alegria M, Duarte CS, Bird HR. Outcomes of serious antisocial behavior from childhood to early adulthood in two Puerto Rican samples in two contexts. Soc Psychiatry Psychiatr Epidemiol 2022; 57: 267–77. 34357404 10.1007/s00127-021-02148-z PMC9923882

[18] Colman I, Murray J, Abbott RA, Maughan B, Kuh D, Croudace TJ, et al. Outcomes of conduct problems in adolescence: 40 year follow-up of national cohort. BMJ 2009; 338: a2981. 19131382 10.1136/bmj.a2981 PMC2615547

[19] Melaku YA, Appleton S, Reynolds AC, Sweetman AM, Stevens DJ, Lack L, et al. Association between childhood behavioral problems and insomnia symptoms in adulthood. JAMA Netw Open 2019; 2(9): e1910861. 31490538 10.1001/jamanetworkopen.2019.10861 PMC6735491

[20] Hoffmann MS, Evans-Lacko S, Collishaw S, Knapp M, Pickles A, Shearer C, et al. Parent- and teacher-reported associations from adolescent bifactor models of psychopathology: an outcome-wide association study of 26 outcomes in mid-life. J Child Psychol Psychiatry 2023; 64: 397–407. 36151972 10.1111/jcpp.13707

[21] Erskine HE, Norman RE, Ferrari AJ, Chan GC, Copeland WE, Whiteford HA, et al. Long-term outcomes of attention-deficit/hyperactivity disorder and conduct disorder: a systematic review and meta-analysis. J Am Acad Child Adolesc Psychiatry 2016; 55: 841–50. 27663939 10.1016/j.jaac.2016.06.016

[22] Thapar AK, Riglin L, Blakey R, Collishaw S, Davey Smith G, et al. Childhood attention-deficit hyperactivity disorder problems and mid-life cardiovascular risk: prospective population cohort study. Br J Psychiatry 2023; 223: 472–7. 37408455 10.1192/bjp.2023.90 PMC7615511

[23] Stringhini S, Carmeli C, Jokela M, Avendaño M, Muennig P, Guida F, et al. Socioeconomic status and the 25 × 25 risk factors as determinants of premature mortality: a multicohort study and meta-analysis of 1.7 million men and women. Lancet 2017; 389: 1229–37. 28159391 10.1016/S0140-6736(16)32380-7 PMC5368415

[24] Stormacq C, Van den Broucke S, Wosinski J. Does health literacy mediate the relationship between socioeconomic status and health disparities? Integrative review. Health Promot Int 2019; 34(5): e1–17. 30107564 10.1093/heapro/day062

[25] Raghupathi V, Raghupathi W. The influence of education on health: an empirical assessment of OECD countries for the period 1995–2015. Arch Public Health 2020; 78: 20. 32280462 10.1186/s13690-020-00402-5 PMC7133023

[26] Zhang X, Lu J, Wu C, Cui J, Wu Y, Hu A, et al. Healthy lifestyle behaviours and all-cause and cardiovascular mortality among 0.9 million Chinese adults. Int J Behav Nutr Phys Act 2021; 18(1): 162. 34922591 10.1186/s12966-021-01234-4 PMC8684211

[27] Opie R, Abbott G, Crawford D, Ball K. Exploring the associations of depressive symptoms with healthy eating self-efficacy over time amongst women in the READI cohort study. Int J Behav Nutr Phys Act 2021; 18(1): 161. 34922558 10.1186/s12966-021-01233-5 PMC8684166

[28] Roshanaei-Moghaddam B, Katon WJ, Russo J. The longitudinal effects of depression on physical activity. Gen Hosp Psychiatry 2009; 31: 306–15. 19555789 10.1016/j.genhosppsych.2009.04.002

[29] Goldstein BI, Carnethon MR, Matthews KA, McIntyre RS, Miller GE, Raghuveer G, et al. Major depressive disorder and bipolar disorder predispose youth to accelerated atherosclerosis and early cardiovascular disease: a scientific statement from the American Heart Association. Circulation 2015; 132: 965–86. 26260736 10.1161/CIR.0000000000000229

[30] Inoue-Choi M, Christensen CH, Rostron BL, Cosgrove CM, Reyes-Guzman C, Apelberg B, et al. Dose-response association of low-intensity and nondaily smoking with mortality in the United States. JAMA Netw Open 2020; 3(6): e206436. 32492162 10.1001/jamanetworkopen.2020.6436 PMC7272118

[31] Khalife N, Kantomaa M, Glover V, Tammelin T, Laitinen J, Ebeling H, et al. Childhood attention-deficit/hyperactivity disorder symptoms are risk factors for obesity and physical inactivity in adolescence. J Am Acad Child Adolesc Psychiatry 2014; 53: 425–36. 24655652 10.1016/j.jaac.2014.01.009

[32] Peters T, Nullig L, Antel J, Naaresh R, Laabs BH, Tegeler L, et al. The role of genetic variation of BMI, body composition, and fat distribution for mental traits and disorders: a look-up and Mendelian randomization study. Front Genet 2020; 11: 373. 32373164 10.3389/fgene.2020.00373 PMC7186862

[33] Thumfart KM, Jawaid A, Bright K, Flachsmann M, Mansuy IM. Epigenetics of childhood trauma: long term sequelae and potential for treatment. Neurosci Biobehav Rev 2022; 132: 1049–66. 34742726 10.1016/j.neubiorev.2021.10.042

[34] Lundgren O, Henriksson P, Delisle Nyström C, Silfvernagel K, Löf M. et al. Hyperactivity is associated with higher fat-free mass and physical activity in Swedish preschoolers: a cross-sectional study. Acta Paediatr 2021; 110: 1273–80. 33020960 10.1111/apa.15608 PMC7984399

[35] Saad SM, Randhawa G, Pang D. Absence of association between behavior problems in childhood and hypertension in midlife. PLoS One 2016; 11(12): e0167831. 27936147 10.1371/journal.pone.0167831 PMC5148005

[36] Kivimaki M, Bartolomucci A, Kawachi I. The multiple roles of life stress in metabolic disorders. Nat Rev Endocrinol 2023; 19: 10–27. 36224493 10.1038/s41574-022-00746-8 PMC10817208

[37] Burford NG, Webster NA, Cruz-Topete D. Hypothalamic-pituitary-adrenal axis modulation of glucocorticoids in the cardiovascular system. Int J Mol Sci 2017; 18(10): 2150. 29035323 10.3390/ijms18102150 PMC5666832

[38] Kuzawa CW, Sweet E. Epigenetics and the embodiment of race: developmental origins of US racial disparities in cardiovascular health. Am J Hum Biol 2009; 21: 2–15. 18925573 10.1002/ajhb.20822

[39] Golden SH, Lazo M, Carnethon M, Bertoni AG, Schreiner PJ, Diez Roux AV, et al. Examining a bidirectional association between depressive symptoms and diabetes. JAMA 2008; 299: 2751–9. 18560002 10.1001/jama.299.23.2751 PMC2648841

[40] von Stumm S, Deary IJ, Kivimäki M, Jokela M, Clark H, Batty GD. Childhood behavior problems and health at midlife: 35-year follow-up of a Scottish birth cohort. J Child Psychol Psychiatry 2011; 52: 992–1001. 21294730 10.1111/j.1469-7610.2011.02373.x

