# Supplementary material for: Childhood psychosocial adjustment and midlife obesity, diabetes and hypertension: prospective study from two birth cohorts
Source: Br J Psychiatry. 2024 Dec;225(6):563–71. doi: 10.1192/bjp.2024.133 (PMC11669467; doi:10.1192/bjp.2024.133)
Supplement: Liu et al. supplementary material [file S0007125024001338sup001.docx]

**Supplementary materials**

**Supplementary method**

**Table S1**. Questions about exposure, mediators, outcomes, and covariates by year and by cohorts

**Table S2**. Compositions of subtypes of behavior by scales and by cohorts

**Table S3.** Participant characteristic by cohorts

**Table S4**. Baseline characteristic according to midlife hypertension status for NCDS

**Table S5**. Baseline characteristic according to midlife diabetes status for NCDS

**Table S6**. Baseline characteristic according to midlife obesity status for NCDS

**Table S7**. Baseline characteristic according to midlife hypertension status for BCS70

**Table S8**. Baseline characteristic according to midlife diabetes status for BCS70

**Table S9**. Baseline characteristic according to midlife obesity status for BCS70

**Table S10**. Sensitivity analysis, association between CPA and the midlife cardiometabolic diseases according to sex for NCDS

**Table S11**. Sensitivity analysis, association between CPA and the midlife cardiometabolic diseases according to sex for BCS70

**Table S12**. Sensitivity analysis, association between CPA and the midlife cardiometabolic diseases according to sex for the NCDS and BSC70 Meta-analyses

**Table S13**. Sensitivity analysis, association between CPA and the midlife cardiometabolic diseases according to social class for NCDS

**Table S14**. Sensitivity analysis, association between CPA and the midlife cardiometabolic diseases according to social class for BCS70

**Table S15**. Sensitivity analysis, association between CPA and the midlife cardiometabolic diseases according to social class for the NCDS and BSC70 Meta-analyses

**Table S16**. Sensitivity analysis, association between CPA and the midlife cardiometabolic diseases with fully imputed data

**Table S17**. Sensitivity analysis, mediation analysis using first tertile of CPA score as dummy variable.

**Table S18**. Sensitivity analysis, mediation analysis using first and second tertiles of CPA score as dummy variable.

**Table S19**. Association between Mediators and midlife cardiometabolic diseases.

**Supplementary method**

**Study population**

The current study utilized data from the 1958 National Child Development Study (NCDS) and the 1970 British Cohort Study (BCS70). The wave conducted at age 10 for NCDS and at age 11 for BCS70 were considered as the baseline. Fixed baseline variables were sourced from earlier waves (NCDS: waves at ages 0 and 7; BCS70: waves at ages 0 and 5). The questions concerning all variables by cohort are presented in **Table S1**.

**Exposure**

In the NCDS, Childhood Psychosocial Adjustment (CPA) was evaluated using the Bristol Social Adjustment Guides (BSAG), which is comprised of 250 behavioral descriptors. This guide posits that certain behavior disturbance 'syndromes' are meaningful, and separate scores can be obtained for each category [1, 2]: 1) Unforthcomingness, 2) Withdrawal, 3) Depression, 4) Anxiety for acceptance by adults, 5) Hostility towards adults, 6) 'Writing off' of adults and adult standards, 7) Anxiety for acceptance by children, 8) Hostility towards children, 9) Restlessness, 10) 'Inconsequential' behaviour, 11) Miscellaneous symptoms, 12) Miscellaneous nervous symptoms.

For BCS70, CPA was measured using the Rutter Child Behaviour Scale, which includes 19 items describing behaviors [3]: 1) Very restless. Often running about or jumping up and down. Hardly ever still, 2) Is squirmy or fidgety, 3) Often destroys own or others’ belongings, 4) Frequently fights with other children, 5) Not much liked by other children, 6) Often worried, worries about many things, 7) Tends to do things on his/her own – rather solitary, 8) Irritable. Is quick to fly off the handle, 9) Often appears miserable, unhappy, tearful, or distressed, 10) Sometimes takes things belonging to others, 11) Has twitches, mannerisms or tics of the face or body, 12) Frequently sucks thumb or finger, 13) Frequently bites nails or fingers, 14) Is often disobedient, 15) Cannot settle to anything for more than a few moments, 16) Tends to be fearful or afraid of new things or new situations, 17) Is over fussy or over particular, 18) Often tells lies, 19) Bullies other children.

These items were further categorized into four CPA subtypes [3], detailed in **Table S2**. Higher scores indicate poorer CPA across all four subtypes, and scores were categorized into tertiles for analysis.

**Mediators**

The study considered educational attainment, smoking habits, and depressive symptoms during young adulthood as mediators. For NCDS, mediators were assessed at ages 23 and 33. 'Ever smoking' was defined as current smoking and self-reported regular smoking. Lower educational attainment was defined as the highest qualification being O level or below. Depressive symptoms were evaluated using the Malaise Inventory, a 24-item scale adapted from the Cornell Medical Index Health Questionnaire; a score of 8 points or higher indicated depression [4]. For BCS70, mediators were assessed at ages 26 and 30, with the same definitions as those applied in the NCDS.

**Covariates**

Covariates included sex, childhood region of residence, parental social class, maternal smoking during pregnancy, method of delivery, birth weight, and breastfeeding history. In NCDS, data on sex, maternal smoking during pregnancy, method of delivery, birth weight, and breastfeeding were collected at ages 0 and 7. For BCS70, these variables were collected at ages 0 and 5, as detailed in **Table S1**.

**References**

1. Stott DH. The social adjustment of children: Manual of the Bristol SocialAdjustment Guides: University of London Press; 1966.

2. Engel M. The Social Adjustment of Children: The Bristol Social Adjustment Guides. *AMA Archives of General Psychiatry*. 1959;1 (5):556-556. doi: 10.1001/archpsyc.1959.03590050124016.

3. Butler NR, and Jean. Golding. From Birth to Five : A Study of the Health and Behaviour of Britain’s 5-Year-Olds: Oxford: Pergamon; 1986.

4. Cheung SY, et al. Malaise scores in adulthood of children and young people who have been in care. *J Child Psychol Psychiatry*. 1997;38 (5):575-580. doi: 10.1111/j.1469-7610.1997.tb01544.x.

**Table S1. Questions about exposure, mediators, outcomes, and covariates by year and by cohorts**

|  |  | NCDS | | BCS70 | |
| --- | --- | --- | --- | --- | --- |
|  | Variables | Age | Question | Age | Question |
| Baseline | Sex | Age 0 | Sex of child | Age 0 | Sex of the Baby |
|  | Smoking during pregnancy | Age 0 | Smoking during pregnancy | Age 0 | Smoking during pregnancy |
|  | Method of delivery | Age 0 | Method of actual delivery | Age 0 | Method of Delivery |
|  | Birth weight | Age 0 | Birthweight (ounces) or estimate | Age 0 | Birthweight of Baby in Grams |
|  | Breastfeeding | Age 7 | Was the child breast fed (partly or wholly) as a baby | Age 5 | Was the child breast fed partly or wholly, even for a few days? |
|  | CPA assessment | Age 11 | Bristol Social Adjustment Guides | Age 10 | Rutter Child Behaviour Scale |
|  | Region of residence | Age 11 | Region at NCDS3 | Age 10 | Region at age 10 follow-up |
|  | Social class | Age 11 | Social class of father or male head | Age 10 | Social class from father’s occupation (or mothers if missing) |
|  | Childhood smoke exposure |  |  | Age 10 | Does the mother/father smoke cigarettes or cigars at present? If mother/father is a non-smoker, has she/he smoked at all in the past ten years? |
| Mediator | Smoking habit | Age 23 | Current cigarette smoking  Ever smoked cigarettes, cigar, or pipe  Ever smoked cigarettes regularly | Age 26 | Which of the following describes your smoking habit |
|  | Educational level | Age 23 | Highest Qualification | Age 26 | Highest Qualification |
|  | Depressive symptom | Age 23 | Malaise Inventory | Age 26 | Malaise Inventory |
|  | Smoking habit | Age 33 | Do you smoke cigarettes at all nowadays  No. cigarettes/day usually smoked  Ever smoked cigarettes regularly | Age 30 | Current smoking status |
|  | Educational level | Age 33 | Highest qualification gained at age 33 | Age 30 | Highest NVQ level |
|  | Depressive symptom | Age 33 | Malaise Inventory | Age 30 | Malaise Inventory |
| Outcome | Hypertension/Diabetes | Age 33 | Have you ever suffered from or been told you had high blood pressure/diabetes | Age 30 | Have you ever had or been told you had high blood pressure/diabetes |
|  | Hypertension/Diabetes |  |  | Age 34 | Since last interview have you had high blood pressure/diabetes |
|  | Hypertension/Diabetes | Age 42 | Have you ever had or been told you had high blood pressure/diabetes | Age 38 | Whether you are currently suffering from high blood pressure/diabetes |
|  | Hypertension/Diabetes | Age 50 | Are you currently suffering from high blood pressure/diabetes | Age 42 | Since last interview have you had high blood pressure/diabetes |
|  | Hypertension/Diabetes | Age 55 | Since last interview have you had high blood pressure/diabetes | Age 46 | Since last interview have you had high blood pressure/diabetes |
|  | Obesity | Age 33 | Height in centimeters  Weight in kilograms | Age 30 | What is your current weight without clothes on  How tall are you without shoes |
|  | Obesity |  |  | Age 34 | What is your current weight without clothes on  How tall are you without shoes |
|  | Obesity | Age 42 | What is your current weight without clothes on  How tall are you without shoes | Age 38 |  |
|  | Obesity | Age 50 | Weight in kilograms | Age 42 | What is your weight without clothes  How tall are you without shoes |
|  | Obesity | Age 55 | What is your weight | Age 46 | What is your weight without clothes  How tall are you without shoes |
| Abbreviations: NCDS, National Childhood Development Study; CPA, childhood psychosocial adjustment | | | | | |

**Table S2. Compositions of subtypes of behavior by scales and by cohorts.**

| Subtype | NCDS | BCS70 |
| --- | --- | --- |
| Conduct problems | Unforthcomingness  Withdrawal  Hostility towards adults  “Writing off” of adults and adult standards  Hostility towards children | Often destroys own or others’ belongings  Frequently fights with other children  Irritable, is quick to fly off the handle  Sometimes takes things belonging to others  Is often disobedient  Often tells lies  Bully other children |
| Emotional problems | Depression Anxiety for acceptance by adults Anxiety for acceptance by children | Often worried, worries about many things  Tends to do things on his/her own, rather solitary  Often appears miserable, unhappy, tearful, or distressed  Tends to be fearful or afraid of new things or new situations  Is fussy or over particular |
| Hyperactivity | Restlessness  Inconsequential behaviour | Very restless. Often running about or jumping up and down. Hardly ever still  Is squirmy or fidgety  Cannot settle to anything for more than a few moments |
| Miscellaneous | Miscellaneous symptoms  Miscellaneous nervous symptoms | Not much liked by other children  Has twitches, mannerisms or tics of the face or body  Frequently sucks thumb or finger  Frequently bites nails or fingers |
| Abbreviations: NCDS, National Childhood Development Study; CPA, childhood psychosocial adjustment | | |

**Table S3. Participant characteristic by cohorts**

|  | NCDS | | | |  | BCS70 | | | |
| --- | --- | --- | --- | --- | --- | --- | --- | --- | --- |
|  | CPA score ^d^ | | | | | | | | |
|  | Overall | Tertile 1 | Tertile 2 | Tertile 3 |  | Overall | Tertile 1 | Tertile 2 | Tertile 3 |
| **Childhood baseline characteristic ^a^** | 14154 | 4718 | 4718 | 4718 |  | 12774 | 4258 | 4258 | 4258 |
| Sex, % |  |  |  |  |  |  |  |  |  |
| Male | 7273 (51.4) | 1988 (42.1) | 2429 (51.5) | 2856 (60.5) |  | 6567 (51.4) | 2031 (47.7) | 2123 (49.9) | 2413 (56.7) |
| Female | 6881 (48.6) | 2730 (57.9) | 2289 (48.5) | 1862 (39.5) |  | 6207 (48.6) | 2227 (52.3) | 2135 (50.1) | 1845 (43.3) |
| Region of residence, % |  |  |  |  |  |  |  |  |  |
| Northern Regions | 5321 (37.6) | 1785 (37.8) | 1732 (36.7) | 1804 (38.2) |  | 4973 (38.9) | 1670 (39.2) | 1681 (39.5) | 1622 (38.1) |
| Central Regions | 4492 (31.7) | 1475 (31.3) | 1513 (32.1) | 1504 (31.9) |  | 2934 (23.0) | 938 (22.0) | 967 (22.7) | 1029 (24.2) |
| Southern Regions | 4341 (30.7) | 1458 (30.9) | 1473 (31.2) | 1410 (29.9) |  | 4835 (37.9) | 1639 (38.5) | 1599 (37.6) | 1597 (37.5) |
| Missing | 0 (0) | 0 (0) | 0 (0) | 0 (0) |  | 32 (0.3) | 11 (0.3) | 11 (0.3) | 10 (0.2) |
| Social class , % |  |  |  |  |  |  |  |  |  |
| I/II | 2796 (19.8) | 745 (15.8) | 899 (19.1) | 1152 (24.4) |  | 3668 (28.7) | 1468 (34.5) | 1256 (29.5) | 944 (22.2) |
| IIIa/b | 6325 (44.7) | 2091 (44.3) | 2166 (45.9) | 2068 (43.8) |  | 6456 (50.5) | 2037 (47.8) | 2188 (51.4) | 2231 (52.4) |
| IV/V | 3525 (24.9) | 1460 (30.9) | 1159 (24.6) | 906 (19.2) |  | 2173 (17.0) | 604 (14.2) | 668 (15.7) | 901 (21.2) |
| Missing | 1508 (10.7) | 422 (8.9) | 494 (10.5) | 592 (12.5) |  | 477 (3.7) | 149 (3.5) | 146 (3.4) | 182 (4.3) |
| Smoking during pregnancy, % |  |  |  |  |  |  |  |  |  |
| No | 8902 (62.9) | 3144 (66.6) | 3018 (64.0) | 2740 (58.1) |  | 6464 (50.6) | 2299 (54.0) | 2184 (51.3) | 1981 (46.5) |
| Any | 4390 (31.0) | 1298 (27.5) | 1428 (30.3) | 1664 (35.3) |  | 5420 (42.4) | 1672 (39.3) | 1778 (41.8) | 1970 (46.3) |
| Missing | 862 (6.1) | 276 (5.8) | 272 (5.8) | 314 (6.7) |  | 890 (7.0) | 287 (6.7) | 296 (7.0) | 307 (7.2) |
| Method of delivery, % |  |  |  |  |  |  |  |  |  |
| Others | 1601 (11.3) | 585 (12.4) | 535 (11.3) | 481 (10.2) |  | 1702 (13.3) | 539 (12.7) | 583 (13.7) | 580 (13.6) |
| Spontaneous | 11857 (83.8) | 3908 (82.8) | 3969 (84.1) | 3980 (84.4) |  | 10211 (79.9) | 3443 (80.9) | 3388 (79.6) | 3380 (79.4) |
| Missing | 696 (4.9) | 225 (4.8) | 214 (4.5) | 257 (5.4) |  | 861 (6.7) | 276 (6.5) | 287 (6.7) | 298 (7.0) |
| Birth weight, kg |  |  |  |  |  |  |  |  |  |
| Mean (SD) | 3.61 (1.89) | 3.60 (1.85) | 3.66 (2.00) | 3.56 (1.82) |  | 3.31 (0.52) | 3.33 (0.52) | 3.32 (0.52) | 3.28 (0.53) |
| Missing | 753 (5.3) | 245 (5.2) | 233 (4.9) | 275 (5.8) |  | 852 (6.7) | 274 (6.4) | 290 (6.8) | 288 (6.8) |
| Breastfeeding, % |  |  |  |  |  |  |  |  |  |
| Never | 3925 (27.7) | 1255 (26.6) | 1280 (27.1) | 1390 (29.5) |  | 6669 (52.2) | 2157 (50.7) | 2208 (51.9) | 2304 (54.1) |
| Any | 8547 (60.4) | 2995 (63.5) | 2883 (61.1) | 2669 (56.6) |  | 3930 (30.8) | 1379 (32.4) | 1329 (31.2) | 1222 (28.7) |
| Missing | 1682 (11.9) | 468 (9.9) | 555 (11.8) | 659 (14.0) |  | 2175 (17.0) | 722 (17.0) | 721 (16.9) | 732 (17.2) |
| Childhood smoke exposure, % |  |  |  |  |  |  |  |  |  |
| No | / | / | / | / |  | 3727 (29.2) | 1389 (32.6) | 1297 (30.5) | 1041 (24.4) |
| Any | / | / | / | / |  | 9045 (70.8) | 2867 (67.3) | 2961 (69.5) | 3217 (75.6) |
| Missing | / | / | / | / |  | 2 (0.0) | 2 (0.0) | 0 (0) | 0 (0) |
| CPA score, point | 5 [2, 13] | 1 [0, 2] | 5 [4, 7] | 17 [13, 23] |  | 421 [289, 571] | 232 [156, 289] | 421 [378, 464] | 645 [571, 752] |
| **Young adulthood Mediators ^b^** |  |  |  |  |  |  |  |  |  |
| Smoking habit, % |  |  |  |  |  |  |  |  |  |
| Never | 7372 (52.1) | 2645 (56.1) | 2481 (52.6) | 2246 (47.6) |  | 6097 (47.7) | 2050 (48.1) | 2040 (47.9) | 2007 (47.1) |
| Ever | 5464 (38.6) | 1708 (36.2) | 1772 (37.6) | 1984 (42.1) |  | 3779 (29.6) | 1295 (30.4) | 1278 (30.0) | 1206 (28.3) |
| Missing | 1318 (9.3) | 365 (7.7) | 465 (9.9) | 488 (10.3) |  | 2898 (22.7) | 913 (21.4) | 940 (22.1) | 1045 (24.5) |
| Educational attainment, % |  |  |  |  |  |  |  |  |  |
| A Level or above | 7543 (53.3) | 2804 (59.4) | 2542 (53.9) | 2197 (46.6) |  | 6643 (52.0) | 2376 (55.8) | 2223 (52.2) | 2044 (48.0) |
| O Level or below | 4535 (32.0) | 1386 (29.4) | 1537 (32.6) | 1612 (34.2) |  | 3461 (27.1) | 1125 (26.4) | 1183 (27.8) | 1153 (27.1) |
| Missing | 2076 (14.7) | 528 (11.2) | 639 (13.5) | 909 (19.3) |  | 2670 (20.9) | 757 (17.8) | 852 (20.0) | 1061 (24.9) |
| Depression, % |  |  |  |  |  |  |  |  |  |
| No | 10797 (76.3) | 3894 (82.5) | 3673 (77.9) | 3230 (68.5) |  | 8414 (65.9) | 3010 (70.7) | 2867 (67.3) | 2537 (59.6) |
| Yes | 1245 (8.8) | 293 (6.2) | 393 (8.3) | 559 (11.8) |  | 1752 (13.7) | 504 (11.8) | 560 (13.2) | 688 (16.2) |
| Missing | 2112 (14.9) | 531 (11.3) | 652 (13.8) | 929 (19.7) |  | 2608 (20.4) | 744 (17.5) | 831 (19.5) | 1033 (24.3) |
| **Midlife cardiometabolic disease ^c^** |  |  |  |  |  |  |  |  |  |
| Hypertension, % |  |  |  |  |  |  |  |  |  |
| No | 8465 (59.8) | 2981 (63.2) | 2906 (61.6) | 2578 (54.6) |  | 8859 (69.4) | 3093 (72.6) | 2971 (69.8) | 2795 (65.6) |
| Yes | 2962 (20.9) | 1018 (21.6) | 940 (19.9) | 1004 (21.3) |  | 1726 (13.5) | 533 (12.5) | 564 (13.2) | 629 (14.8) |
| Missing | 2727 (19.3) | 719 (15.2) | 872 (18.5) | 1136 (24.1) |  | 2189 (17.1) | 632 (14.8) | 723 (17.0) | 834 (19.6) |
| Diabetes, % |  |  |  |  |  |  |  |  |  |
| No | 10767 (76.1) | 3811 (80.8) | 3633 (77.0) | 3323 (70.4) |  | 10178 (79.7) | 3510 (82.4) | 3390 (79.6) | 3278 (77.0) |
| Yes | 660 (4.7) | 187 (4.0) | 213 (4.5) | 260 (5.5) |  | 407 (3.2) | 116 (2.7) | 145 (3.4) | 146 (3.4) |
| Missing | 2727 (19.3) | 720 (15.3) | 872 (18.5) | 1135 (24.1) |  | 2189 (17.1) | 632 (14.8) | 723 (17.0) | 834 (19.6) |
| Obesity, % |  |  |  |  |  |  |  |  |  |
| No | 7469 (52.8) | 2748 (58.2) | 2514 (53.3) | 2207 (46.8) |  | 7463 (58.4) | 2628 (61.7) | 2499 (58.7) | 2336 (54.9) |
| Yes | 3537 (25.0) | 1143 (24.2) | 1188 (25.2) | 1206 (25.6) |  | 2727 (21.3) | 886 (20.8) | 922 (21.7) | 919 (21.6) |
| Missing | 3148 (22.2) | 827 (17.5) | 1016 (21.5) | 1305 (27.7) |  | 2584 (20.2) | 744 (17.5) | 837 (19.7) | 1003 (23.6) |
| Abbreviations: NCDS, National Childhood Development Study; BCS70, 1970 British Cohort Study, CPA, childhood psychosocial adjustment.  ^a^ Collected at age 10 on NCDS and age 11 on BCS70.  ^b^ Collected at ages 23, 33 on NCDS and ages 26, 30 on BCS 70.  ^c^ Collected at ages 33, 42, 50, 55 on NCDS and ages 30, 34, 38, 42, 46 on BCS70.  ^d^ Assessed at age 10 on NCDS by Bristol Social Adjustment Guides and age 11 on BCS70 by Rutter Child Behavior Scale. | | | | | | | | | |

**Table S4. Baseline characteristic according to midlife hypertension status for NCDS.**

|  | Overall | Without hypertension | With hypertension |
| --- | --- | --- | --- |
|  | 11427 | 8465 | 2962 |
| Sex, % |  |  |  |
| Male | 5744 (50.3) | 4272 (50.5) | 1472 (49.7) |
| Female | 5683 (49.7) | 4193 (49.5) | 1490 (50.3) |
| Region of residence, % |  |  |  |
| Northern Regions | 4255 (37.2) | 3165 (37.4) | 1090 (36.8) |
| Central Regions | 3704 (32.4) | 2723 (32.2) | 981 (33.1) |
| Southern Regions | 3468 (30.3) | 2577 (30.4) | 891 (30.1) |
| Social class, % |  |  |  |
| I/II | 2226 (19.5) | 1603 (18.9) | 623 (21.0) |
| IIIa/b | 5179 (45.3) | 3796 (44.8) | 1383 (46.7) |
| IV/V | 2938 (25.7) | 2249 (26.6) | 689 (23.3) |
| Missing | 1084 (9.5) | 817 (9.7) | 267 (9.0) |
| Smoking during pregnancy, % |  |  |  |
| No | 7246 (63.4) | 5467 (64.6) | 1779 (60.1) |
| Any | 3569 (31.2) | 2552 (30.1) | 1017 (34.3) |
| Missing | 612 (5.4) | 446 (5.3) | 166 (5.6) |
| Method of delivery, % |  |  |  |
| Others | 1309 (11.5) | 956 (11.3) | 353 (11.9) |
| Spontaneous | 9639 (84.4) | 7162 (84.6) | 2477 (83.6) |
| Missing | 479 (4.2) | 347 (4.1) | 132 (4.5) |
| Birth weight, kg |  |  |  |
| Mean (SD) | 3.60 (1.87) | 3.62 (1.87) | 3.54 (1.85) |
| Missing, % | 521 (4.6) | 379 (4.5) | 142 (4.8) |
| Breastfeeding, % |  |  |  |
| Never | 3195 (28.0) | 2363 (27.9) | 832 (28.1) |
| Any | 7033 (61.5) | 5222 (61.7) | 1811 (61.1) |
| Missing | 1199 (10.5) | 880 (10.4) | 319 (10.8) |
| CPA score, point | 5 [2, 12] | 5 [1, 12] | 5 [2, 13] |
| CPA Tertile, % |  |  |  |
| Tertile 1 | 3809 (33.3) | 2837 (33.5) | 972 (32.8) |
| Tertile 2 | 3809 (33.3) | 2888 (34.1) | 921 (31.1) |
| Tertile 3 | 3809 (33.3) | 2740 (32.4) | 1069 (36.1) |
| Abbreviations: NCDS, National Childhood Development Study; CPA, childhood psychosocial adjustment | | | |

**Table S5. Baseline characteristic according to midlife diabetes status for NCDS**

|  | Overall | Without diabetes | With diabetes |
| --- | --- | --- | --- |
|  | 11427 | 10767 | 660 |
| Sex, % |  |  |  |
| Male | 5744 (50.3) | 5386 (50.0) | 358 (54.2) |
| Female | 5683 (49.7) | 5381 (50.0) | 302 (45.8) |
| Region of residence, % |  |  |  |
| Northern Regions | 4255 (37.2) | 4008 (37.2) | 247 (37.4) |
| Central Regions | 3704 (32.4) | 3490 (32.4) | 214 (32.4) |
| Southern Regions | 3468 (30.3) | 3269 (30.4) | 199 (30.2) |
| Social class, % |  |  |  |
| I/II | 2227 (19.5) | 2074 (19.3) | 153 (23.2) |
| IIIa/b | 5178 (45.3) | 4870 (45.2) | 308 (46.7) |
| IV/V | 2938 (25.7) | 2795 (26.0) | 143 (21.7) |
| Missing | 1084 (9.5) | 1028 (9.5) | 56 (8.5) |
| Smoking during pregnancy, % |  |  |  |
| No | 7246 (63.4) | 6875 (63.9) | 371 (56.2) |
| Any | 3569 (31.2) | 3318 (30.8) | 251 (38.0) |
| Missing | 612 (5.4) | 574 (5.3) | 38 (5.8) |
| Method of delivery, % |  |  |  |
| Others | 1309 (11.5) | 1232 (11.4) | 77 (11.7) |
| Spontaneous | 9639 (84.4) | 9088 (84.4) | 551 (83.5) |
| Missing | 479 (4.2) | 447 (4.2) | 32 (4.8) |
| Birth weight, kg |  |  |  |
| Mean (SD) | 3.60 (1.87) | 3.61 (1.88) | 3.43 (1.60) |
| Missing, % | 521 (4.6) | 488 (4.5) | 33 (5.0) |
| Breastfeeding, % |  |  |  |
| Never | 3195 (28.0) | 3008 (27.9) | 187 (28.3) |
| Any | 7033 (61.5) | 6625 (61.5) | 408 (61.8) |
| Missing | 1199 (10.5) | 1134 (10.5) | 65 (9.8) |
| CPA score, point | 5 [2, 12] | 5 [1, 12] | 7 [2, 15] |
| CPA Tertile, % |  |  |  |
| Tertile 1 | 3809 (33.3) | 3629 (33.7) | 180 (27.3) |
| Tertile 2 | 3809 (33.3) | 3609 (33.5) | 200 (30.3) |
| Tertile 3 | 3809 (33.3) | 3529 (32.8) | 280 (42.4) |
| Abbreviations: NCDS, National Childhood Development Study; CPA, childhood psychosocial adjustment | | | |

**Table S6. Baseline characteristic according to midlife obesity status for NCDS**

|  | Overall | Not obesity | Obesity |
| --- | --- | --- | --- |
|  | 11006 | 7469 | 3537 |
| Sex, % |  |  |  |
| Male | 5511 (50.1) | 3686 (49.4) | 1825 (51.6) |
| Female | 5495 (49.9) | 3783 (50.6) | 1712 (48.4) |
| Region of residence, % |  |  |  |
| Northern Regions | 4105 (37.3) | 2795 (37.4) | 1310 (37.0) |
| Central Regions | 3585 (32.6) | 2375 (31.8) | 1210 (34.2) |
| Southern Regions | 3316 (30.1) | 2299 (30.8) | 1017 (28.8) |
| Social class, % |  |  |  |
| I/II | 2152 (19.6) | 1405 (18.8) | 747 (21.1) |
| IIIa/b | 4984 (45.3) | 3278 (43.9) | 1706 (48.2) |
| IV/V | 2830 (25.7) | 2065 (27.6) | 765 (21.6) |
| Missing | 1040 (9.4) | 721 (9.7) | 319 (9.0) |
| Smoking during pregnancy, % |  |  |  |
| No | 6993 (63.5) | 4853 (65.0) | 2140 (60.5) |
| Any | 3433 (31.2) | 2220 (29.7) | 1213 (34.3) |
| Missing | 580 (5.3) | 396 (5.3) | 184 (5.2) |
| Method of delivery, % |  |  |  |
| Others | 1257 (11.4) | 820 (11.0) | 437 (12.4) |
| Spontaneous | 9295 (84.5) | 6333 (84.8) | 2962 (83.7) |
| Missing | 454 (4.1) | 316 (4.2) | 138 (3.9) |
| Birth weight, kg |  |  |  |
| Mean (SD) | 3.60 (1.86) | 3.59 (1.87) | 3.61 (1.85) |
| Missing, % | 495 (4.5%) | 348 (4.7%) | 147 (4.2%) |
| Breastfeeding, % |  |  |  |
| Never | 3076 (27.9) | 2018 (27.0) | 1058 (29.9) |
| Any | 6788 (61.7) | 4659 (62.4) | 2129 (60.2) |
| Missing | 1142 (10.4) | 792 (10.6) | 350 (9.9) |
| CPA score, point | 5 [1, 12] | 5 [1, 11] | 6 [2, 13] |
| CPA Tertile, % |  |  |  |
| Tertile 1 | 3669 (33.3) | 2598 (34.8) | 1071 (30.3) |
| Tertile 2 | 3669 (33.3) | 2501 (33.5) | 1168 (33.0) |
| Tertile 3 | 3668 (33.3) | 2370 (31.7) | 1298 (36.7) |
| Abbreviations: NCDS, National Childhood Development Study; CPA, childhood psychosocial adjustment | | | |

**Table S7. Baseline characteristic according to midlife hypertension status for BCS70**

|  | Overall | Without hypertension | With hypertension |
| --- | --- | --- | --- |
|  | 10585 | 8859 | 1726 |
| Sex, % |  |  |  |
| Male | 5225 (49.4) | 4388 (49.5) | 837 (48.5) |
| Female | 5360 (50.6) | 4471 (50.5) | 889 (51.5) |
| Region of residence, % |  |  |  |
| Northern Regions | 4051 (38.3) | 3438 (38.8) | 613 (35.5) |
| Central Regions | 2484 (23.5) | 2071 (23.4) | 413 (23.9) |
| Southern Regions | 4026 (38.0) | 3329 (37.6) | 697 (40.4) |
| Missing | 24 (0.2) | 21 (0.2) | 3 (0.2) |
| Social class, % |  |  |  |
| I/II | 3131 (29.6) | 2679 (30.2) | 452 (26.2) |
| IIIa/b | 5317 (50.2) | 4451 (50.2) | 866 (50.2) |
| IV/V | 1751 (16.5) | 1422 (16.1) | 329 (19.1) |
| Missing | 386 (3.6) | 307 (3.5) | 79 (4.6) |
| Smoking during pregnancy, % |  |  |  |
| No | 5473 (51.7) | 4649 (52.5) | 824 (47.7) |
| Any | 4425 (41.8) | 3615 (40.8) | 810 (46.9) |
| Missing | 687 (6.5) | 595 (6.7) | 92 (5.3) |
| Method of delivery, % |  |  |  |
| Others | 1428 (13.5) | 1170 (13.2) | 258 (14.9) |
| Spontaneous | 8497 (80.3) | 7122 (80.4) | 1375 (79.7) |
| Missing | 660 (6.2) | 567 (6.4) | 93 (5.4) |
| Birth weight, kg |  |  |  |
| Mean (SD) | 3.31 (0.52) | 3.32 (0.52) | 3.27 (0.52) |
| Missing | 651 (6.2) | 561 (6.3) | 90 (5.2) |
| Breastfeeding, % |  |  |  |
| Never | 5532 (52.3) | 4587 (51.8) | 945 (54.8) |
| Any | 3416 (32.3) | 2898 (32.7) | 518 (30.0) |
| Missing | 1637 (15.5) | 1374 (15.5) | 263 (15.2) |
| Childhood smoke exposure, % |  |  |  |
| No | 3111 (29.4) | 2681 (30.3) | 430 (24.9) |
| Any | 7472 (70.6) | 6177 (69.7) | 1295 (75.0) |
| Missing | 2 (0.0) | 1 (0.0) | 1 (0.1) |
| CPA score, point | 416 [285, 566] | 412 [281, 559] | 439 [301, 598.75] |
| CPA Tertile, % |  |  |  |
| Tertile 1 | 3529 (33.3) | 3010 (34.0) | 519 (30.1) |
| Tertile 2 | 3528 (33.3) | 2973 (33.6) | 555 (32.2) |
| Tertile 3 | 3528 (33.3) | 2876 (32.5) | 652 (37.8) |
| Abbreviations: BCS70, 1970 British Cohort Study, CPA, childhood psychosocial adjustment. | | | |

**Table S8. Baseline characteristic according to midlife diabetes status for BCS70**

|  | Overall | Without diabetes | With diabetes |
| --- | --- | --- | --- |
|  | 10585 | 10178 | 407 |
| Sex, % |  |  |  |
| Male | 5225 (49.4) | 5005 (49.2) | 220 (54.1) |
| Female | 5360 (50.6) | 5173 (50.8) | 187 (45.9) |
| Region of residence, % |  |  |  |
| Northern Regions | 4051 (38.3) | 3907 (38.4) | 144 (35.4) |
| Central Regions | 2484 (23.5) | 2373 (23.3) | 111 (27.3) |
| Southern Regions | 4026 (38.0) | 3876 (38.1) | 150 (36.9) |
| Missing | 24 (0.2) | 22 (0.2) | 2 (0.5) |
| Social class, % |  |  |  |
| I/II | 3131 (29.6) | 3029 (29.8) | 102 (25.1) |
| IIIa/b | 5317 (50.2) | 5108 (50.2) | 209 (51.4) |
| IV/V | 1751 (16.5) | 1670 (16.4) | 81 (19.9) |
| Missing | 386 (3.6) | 371 (3.6) | 15 (3.7) |
| Smoking during pregnancy, % |  |  |  |
| No | 5473 (51.7) | 5279 (51.9) | 194 (47.7) |
| Any | 4425 (41.8) | 4243 (41.7) | 182 (44.7) |
| Missing | 687 (6.5) | 656 (6.4) | 31 (7.6) |
| Method of delivery, % |  |  |  |
| Others | 1428 (13.5) | 1377 (13.5) | 51 (12.5) |
| Spontaneous | 8497 (80.3) | 8170 (80.3) | 327 (80.3) |
| Missing | 660 (6.2) | 631 (6.2) | 29 (7.1) |
| Birth weight, kg |  |  |  |
| Mean (SD) | 3.31 (0.52) | 3.32 (0.52) | 3.23 (0.54) |
| Missing | 651 (6.2) | 622 (6.1) | 29 (7.1) |
| Breastfeeding, % |  |  |  |
| Never | 5532 (52.3) | 5330 (52.4) | 202 (49.6) |
| Any | 3416 (32.3) | 3290 (32.3) | 126 (31.0) |
| Missing | 1637 (15.5) | 1558 (15.3) | 79 (19.4) |
| Childhood smoke exposure, % |  |  |  |
| No | 3111 (29.4) | 2993 (29.4) | 118 (29.0) |
| Any | 7472 (70.6) | 7183 (70.6) | 289 (71.0) |
| Missing | 2 (0.0) | 2 (0.0) | 0 (0.0) |
| CPA score, point | 416 [285, 566] | 416 [284, 564] | 427 [310, 608.50] |
| CPA Tertile, % |  |  |  |
| Tertile 1 | 3529 (33.3) | 3415 (33.6) | 114 (28.0) |
| Tertile 2 | 3528 (33.3) | 3385 (33.3) | 143 (35.1) |
| Tertile 3 | 3528 (33.3) | 3378 (33.2) | 150 (36.9) |
| Abbreviations: BCS70, 1970 British Cohort Study, CPA, childhood psychosocial adjustment. | | | |

**Table S9. Baseline characteristic according to midlife obesity status for BCS70**

|  | Before imputation | | |
| --- | --- | --- | --- |
|  | Overall | Not obesity | Obesity |
|  | 10190 | 7463 | 2727 |
| Sex, % |  |  |  |
| Male | 5035 (49.4) | 3645 (48.8) | 1390 (51.0) |
| Female | 5155 (50.6) | 3818 (51.2) | 1337 (49.0) |
| Region of residence, % |  |  |  |
| Northern Regions | 3892 (38.2) | 2850 (38.2) | 1042 (38.2) |
| Central Regions | 2393 (23.5) | 1699 (22.8) | 694 (25.4) |
| Southern Regions | 3882 (38.1) | 2897 (38.8) | 985 (36.1) |
| Missing | 23 (0.2) | 17 (0.2) | 6 (0.2) |
| Social class, % |  |  |  |
| I/II | 3048 (29.9) | 2370 (31.8) | 678 (24.9) |
| IIIa/b | 5119 (50.2) | 3682 (49.3) | 1437 (52.7) |
| IV/V | 1661 (16.3) | 1152 (15.4) | 509 (18.7) |
| Missing | 362 (3.6) | 259 (3.5) | 103 (3.8) |
| Smoking during pregnancy, % |  |  |  |
| No | 5307 (52.1) | 4012 (53.8) | 1295 (47.5) |
| Any | 4220 (41.4) | 2950 (39.5) | 1270 (46.6) |
| Missing | 663 (6.5) | 501 (6.7) | 162 (5.9) |
| Method of delivery, % |  |  |  |
| Others | 1371 (13.5) | 987 (13.2) | 384 (14.1) |
| Spontaneous | 8183 (80.3) | 5994 (80.3) | 2189 (80.3) |
| Missing | 636 (6.2) | 482 (6.5) | 154 (5.6) |
| Birth weight, kg |  |  |  |
| Mean (SD) | 3.32 (0.52) | 3.31 (0.52) | 3.34 (0.53) |
| Missing | 627 (6.2) | 474 (6.4) | 153 (5.6) |
| Breastfeeding, % |  |  |  |
| Never | 5303 (52.0) | 3853 (51.6) | 1450 (53.2) |
| Any | 3331 (32.7) | 2460 (33.0) | 871 (31.9) |
| Missing | 1556 (15.3) | 1150 (15.4) | 406 (14.9) |
| Childhood smoke exposure, % |  |  |  |
| No | 3010 (29.5) | 2301 (30.8) | 709 (26.0) |
| Any | 7178 (70.4) | 5160 (69.1) | 2018 (74.0) |
| Missing | 2 (0.0) | 2 (0.0) | 0 (0.0) |
| CPA score, point | 414 [284, 562.75] | 410 [282, 559] | 424 [287, 574] |
| CPA Tertile, % |  |  |  |
| Tertile 1 | 3397 (33.3) | 2536 (34.0) | 861 (31.6) |
| Tertile 2 | 3397 (33.3) | 2485 (33.3) | 912 (33.4) |
| Tertile 3 | 3396 (33.3) | 2442 (32.7) | 954 (35.0) |
| Abbreviations: BCS70, 1970 British Cohort Study, CPA, childhood psychosocial adjustment. | | | |

**Table S10. Sensitivity analysis, association between CPA and the midlife cardiometabolic diseases according to sex for NCDS**

| Outcome | Sex | Odd ratio (95% confidence interval) of CPA score tertile | | |
| --- | --- | --- | --- | --- |
|  |  | Tertile 1 | Tertile 2 | Tertile 3 |
| Hypertension | Male | Ref | 0.94 (0.81, 1.09) | 1.07 (0.93, 1.24) |
|  | Female | Ref | 1.00 (0.86, 1.16) | 1.25 (1.08, 1.44) |
|  |  | Tertile 1 | Tertile 2 | Tertile 3 |
| Diabetes | Male | Ref | 1.02 (0.78, 1.33) | 1.24 (0.96, 1.62) |
|  | Female | Ref | 1.00 (0.74, 1.36) | 1.56 (1.17, 2.07) |
|  |  | Tertile 1 | Tertile 2 | Tertile 3 |
| Obesity | Male | Ref | 1.00 (0.87, 1.15) | 1.16 (1.01, 1.33) |
|  | Female | Ref | 1.16 (1.01, 1.34) | 1.34 (1.16, 1.55) |
| Abbreviations: NCDS, National Childhood Development Study; CPA, childhood psychosocial adjustment.  Models were adjusted for region of residence, social class, and smoking during pregnancy, method of delivery, birth weight and breastfeeding。  Sex-specific tertiles were used. | | | | |

**Table S11. Sensitivity analysis, association between CPA and the midlife cardiometabolic diseases according to sex for BCS70**

| Outcome | Sex | Odd ratio (95% confidence interval) of CPA score tertile | | |
| --- | --- | --- | --- | --- |
|  |  | Tertile 1 | Tertile 2 | Tertile 3 |
| Hypertension | Male | Ref | 0.97 (0.81, 1.17) | 1.16 (0.96, 1.39) |
|  | Female | Ref | 1.16 (0.97, 1.39) | 1.28 (1.07, 1.53) |
|  |  | Tertile 1 | Tertile 2 | Tertile 3 |
| Diabetes | Male | Ref | 1.15 (0.83, 1.60) | 1.03 (0.73, 1.44) |
|  | Female | Ref | 1.53 (1.05, 2.22) | 1.50 (1.03, 2.19) |
|  |  | Tertile 1 | Tertile 2 | Tertile 3 |
| Obesity | Male | Ref | 1.03 (0.89, 1.20) | 1.04 (0.89, 1.21) |
|  | Female | Ref | 1.09 (0.93, 1.27) | 1.12 (0.96, 1.31) |
| Abbreviations: BCS70, 1970 British Cohort Study; CPA, childhood psychosocial adjustment.  Models were adjusted for region of residence, social class, and smoking during pregnancy, method of delivery, birth weight, breastfeeding, and childhood smoke exposure.  Sex-specific tertiles were used. | | | | |

**Table S12. Sensitivity analysis, association between CPA and the midlife cardiometabolic diseases according to sex for the NCDS and BSC70 Meta-analyses**

| Outcome | Sex | Odd ratio (95% confidence interval) of CPA score tertile | | |
| --- | --- | --- | --- | --- |
|  |  | Tertile 1 | Tertile 2 | Tertile 3 |
| Hypertension | Male | Ref | 0.95 (0.85, 1.07) | 1.10 (1.00, 1.24) |
|  | Female | Ref | 1.06 (0.95, 1.19) | 1.26 (1.12, 1.41) |
|  |  | Tertile 1 | Tertile 2 | Tertile 3 |
| Diabetes | Male | Ref | 1.07 (0.87, 1.32) | 1.16 (0.94, 1.43) |
|  | Female | Ref | 1.18 (0.93, 1.50) | 1.54 (1.23, 1.93) |
|  |  | Tertile 1 | Tertile 2 | Tertile 3 |
| Obesity | Male | Ref | 1.01 (0.92, 1.12) | 1.10 (1.00, 1.22) |
|  | Female | Ref | 1.13 (1.02, 1.25) | 1.23 (1.11, 1.37) |
| Abbreviations: NCDS, National Childhood Development Study; CPA, childhood psychosocial adjustment.  Fixed effects were considered across studies. All P values for Heterogeneity > 0.1. | | | | |

**Table S13. Sensitivity analysis, association between CPA and the midlife cardiometabolic diseases according to social class for NCDS**

| Outcome | Social class | Odd ratio (95% confidence interval) of CPA score tertile | | |
| --- | --- | --- | --- | --- |
|  |  | Tertile 1 | Tertile 2 | Tertile 3 |
| Hypertension | I/II | Ref | 1.11 (0.89, 1.38) | 1.12 (0.90, 1.40) |
|  | IIIa/b | Ref | 0.91 (0.79, 1.05) | 1.09 (0.95, 1.27) |
|  | IV/V | Ref | 0.86 (0.70, 1.05) | 1.04 (0.84, 1.28) |
|  |  | Tertile 1 | Tertile 2 | Tertile 3 |
| Diabetes | I/II | Ref | 1.27 (0.83, 1.94) | 1.59 (1.07, 2.36) |
|  | IIIa/b | Ref | 1.09 (0.82, 1.46) | 1.47 (1.12, 1.93) |
|  | IV/V | Ref | 1.19 (0.77, 1.83) | 1.50 (1.00, 2.25) |
|  |  | Tertile 1 | Tertile 2 | Tertile 3 |
| Obesity | I/II | Ref | 1.02 (0.82, 1.28) | 1.28 (1.02, 1.60) |
|  | IIIa/b | Ref | 1.13 (0.98, 1.30) | 1.21 (1.05, 1.40) |
|  | IV/V | Ref | 1.15 (0.94, 1.41) | 1.30 (1.06, 1.60) |
| Abbreviations: NCDS, National Childhood Development Study; CPA, childhood psychosocial adjustment.  Models were adjusted for sex, region of residence, and smoking during pregnancy, method of delivery, birth weight and breastfeeding  Social class-specific tertiles were used. | | | | |

**Table S14. Sensitivity analysis, association between CPA and the midlife cardiometabolic diseases according to social class for BCS70**

| Outcome | Social class | Odd ratio (95% confidence interval) of CPA score tertile | | |
| --- | --- | --- | --- | --- |
|  |  | Tertile 1 | Tertile 2 | Tertile 3 |
| Hypertension | I/II | Ref | 1.02 (0.80, 1.29) | 1.00 (0.78, 1.27) |
|  | IIIa/b | Ref | 1.16 (0.97, 1.39) | 1.34 (1.12, 1.59) |
|  | IV/V | Ref | 0.81 (0.60, 1.10) | 1.37 (1.02, 1.84) |
|  |  | Tertile 1 | Tertile 2 | Tertile 3 |
| Diabetes | I/II | Ref | 1.08 (0.67, 1.74) | 1.04 (0.64, 1.70) |
|  | IIIa/b | Ref | 1.42 (0.96, 2.00) | 1.26 (0.89, 1.79) |
|  | IV/V | Ref | 0.74 (0.42, 1.31) | 1.21 (0.72, 2.03) |
|  |  | Tertile 1 | Tertile 2 | Tertile 3 |
| Obesity | I/II | Ref | 1.05 (0.84, 1.30) | 1.14 (0.93, 1.41) |
|  | IIIa/b | Ref | 0.99 (0.85, 1.15) | 1.03 (0.89, 1.20) |
|  | IV/V | Ref | 1.07 (0.83, 1.39) | 1.08 (0.84, 1.41) |
| Abbreviations: BCS70, 1970 British Cohort Study; CPA, childhood psychosocial adjustment.  Models were adjusted for sex, region of residence, smoking during pregnancy, method of delivery, birth weight, breastfeeding, and childhood smoke exposure.  Social class-specific tertiles were used. | | | | |

**Table S15. Sensitivity analysis, association between CPA and the midlife cardiometabolic diseases according to social class for the NCDS and BSC70 Meta-analyses**

| Outcome | Social class | Odd ratio (95% confidence interval) of CPA score tertile | | |
| --- | --- | --- | --- | --- |
|  |  | Tertile 1 | Tertile 2 | Tertile 3 |
| Hypertension | I/II | Ref | 0.98 (0.83, 1.16) | 1.08 (0.91, 1.27) |
|  | IIIa/b | Ref | 1.01 (0.90, 1.14) | 1.20 (1.07, 1.34) |
|  | IV/V | Ref | 0.90 (0.76, 1.07) | 1.18 (1.00, 1.40) |
|  |  | Tertile 1 | Tertile 2 | Tertile 3 |
| Diabetes | I/II | Ref | 1.11 (0.80, 1.54) | 1.39 (1.01, 1.90) |
|  | IIIa/b | Ref | 1.18 (0.95, 1.48) | 1.38 (1.10, 1.72) |
|  | IV/V | Ref | 1.10 (0.79, 1.55) | 1.40 (1.01, 1.94) |
|  |  | Tertile 1 | Tertile 2 | Tertile 3 |
| Obesity | I/II | Ref | 1.06 (0.91, 1.23) | 1.25 (1.07, 1.46) |
|  | IIIa/b | Ref | 1.08 (0.97, 1.19) | 1.13 (1.02, 1.25) |
|  | IV/V | Ref | 1.11 (0.95, 1.30) | 1.19 (1.01, 1.40) |
| Abbreviations: NCDS, National Childhood Development Study; CPA, childhood psychosocial adjustment.  Fixed effects were considered across studies. All P values for Heterogeneity > 0.1. | | | | |

**Table S16. Sensitivity analysis, association between CPA and the midlife cardiometabolic diseases with fully imputed data**

|  | Odd ratio (95% confidence interval) of CPA score tertile | | |
| --- | --- | --- | --- |
|  | BCS70 (N = 12774) ^a^ | NCDS (N = 14154) ^b^ | Meta (N = 26928) |
| CPA score, point | Hypertension | | |
| Tertile 1 | Ref | Ref | Ref |
| Tertile 2 | 1.07 (0.94, 1.21) | 0.95 (0.86, 1.06) | 1.00 (0.92, 1.08) |
| Tertile 3 | 1.19 (1.05, 1.35) | 1.10 (1.00, 1.22) | 1.13 (1.05, 1.23) |
| CPA score, point | Diabetes | | |
| Tertile 1 | Ref | Ref | Ref |
| Tertile 2 | 1.22 (0.95, 1.57) | 1.15 (0.94, 1.40) | 1.17 (1.00, 1.37) |
| Tertile 3 | 1.22 (0.94, 1.60) | 1.39 (1.14, 1.68) | 1.33 (1.14, 1.55) |
| CPA score, point | Obesity | | |
| Tertile 1 | Ref | Ref | Ref |
| Tertile 2 | 1.06 (0.95, 1.18) | 1.08 (0.98, 1.19) | 1.07 (1.00, 1.15) |
| Tertile 3 | 1.07 (0.96, 1.19) | 1.22 (1.10, 1.36) | 1.14 (1.06, 1.23) |
| Abbreviations: NCDS, National Childhood Development Study; CPA, childhood psychosocial adjustment.  ^a^ Models were adjusted for sex, region of residence, social class, and smoking during pregnancy, method of delivery, birth weight and breastfeeding.  ^b^ Models were adjusted for sex, region of residence, social class, and smoking during pregnancy, method of delivery, birth weight, breastfeeding, and childhood smoke exposure. | | | |

**Table S17. Sensitivity analysis, mediation analysis using first tertile of CPA score as dummy variable.**

| Outcome | Mediators | Mediation proportion (95% Confidence interval) | | |
| --- | --- | --- | --- | --- |
|  | | NCDS |  | BCS70 |
| Hypertension | Smoking habit ^a^ | 1.5 (-7.2, 9.3) |  | 0.6 (-0.7, 4.0) |
|  | Educational levels ^b^ | 3.3 (-7.0, 6.2) |  | 2.7 (0.2, 10.0) ^*^ |
|  | Depression ^c^ | 4.1 (-6.3, 11.3) |  | 14.0 (6.2, 46.0) ^**^ |
| Diabetes | Smoking habit ^a^ | 0.2 (-6.1, 7.1) |  | -0.1 (-8.0, 9.0) |
|  | Educational levels ^b^ | 10.0 (2.0, 34.0) ^*^ |  | -0.5 (-6.3, 4.0) |
|  | Depression ^c^ | 14.7 (5.8, 49.0) ^**^ |  | 12.2 (2.9, 56.0) ^*^ |
| Obesity | Smoking habit ^a^ | -0.1 (-4.3, 3.0) |  | -1.6 (-11.7, 10.0) |
|  | Educational levels ^b^ | 17.3 (9.5, 33.0) ^**^ |  | 8.0 (1.9, 35.0) ^*^ |
|  | Depression ^c^ | 4.2 (0.2,11.0) ^*^ |  | 9.4 (3.1, 46.0) ^**^ |
| Abbreviations: NCDS, National Childhood Development Study; BCS70, 1970 British Cohort Study.  ^a^ Ever smoking vs never smoking  ^b^ Highest educational level O level or below vs A level or above  ^c^ With depression vs. without depression  ^*^ P value < 0.05, ^**^ P value < 0.01 | | | | |

**Table S18. Sensitivity analysis, mediation analysis using first and second tertiles of CPA score as dummy variable.**

| Outcome | Mediators | Mediation proportion (95% Confidence interval) | | |
| --- | --- | --- | --- | --- |
|  | | NCDS |  | BCS70 |
| Hypertension | Smoking habit ^a^ | 0.2 (-4.9, 6.0) |  | 0.5 (-0.5, 3.0) |
|  | Educational levels ^b^ | 1.5 (0.6, 3.3) ^**^ |  | 1.6 (0.1, 5.0) ^*^ |
|  | Depression ^c^ | 2.4 (1.2, 5.0) ^**^ |  | 13.8 (7.2, 29.0) ^**^ |
| Diabetes | Smoking habit ^a^ | -0.2 (-4.5, 4.0) |  | -0.2 (-29.0, 28.0) |
|  | Educational levels ^b^ | 6.4 (1.2, 15.0) ^*^ |  | -0.4 (-14.1, 14.0) |
|  | Depression ^c^ | 12.2 (5.4, 25.0) ^**^ |  | 24.1 (-17.8, 29.4) |
| Obesity | Smoking habit ^a^ | -0.1 (-5.7, 3.0) |  | -1.9 (-11.0, 9.6) |
|  | Educational levels ^b^ | 14.6 (8.4, 26.0) ^**^ |  | 9.1 (-4.0, 8.5) |
|  | Depression ^c^ | 3.9 (-0.1,10.0) |  | 16.0 (-3.7, 68.0) |
| Abbreviations: NCDS, National Childhood Development Study; BCS70, 1970 British Cohort Study.  ^a^ Ever smoking vs never smoking  ^b^ Highest educational level O level or below vs A level or above  ^c^ Depression vs. not depression  ^*^ P value < 0.05, ^**^ P value < 0.01 | | | | |

Table S19. Association between Mediators and midlife cardiometabolic diseases

|  |  | Odd ratio (95% Confidence Interval) | | | | | |
| --- | --- | --- | --- | --- | --- | --- | --- |
|  |  | NCDS | | | | | |
| Mediators | Subgroups | Hypertension | P-int | Diabetes | P-int | Obesity | P-int |
| Educational  levels | Overall | 1.20 (1.11, 1.30 |  | 1.17 (1.02, 1.35) |  | 1.30 (1.21, 1.40) |  |
|  |  |  |  |  |  |  |  |
|  | Ever smoking | 1.16 (1.04, 1.29) | 0.33 | 1.07 (0.89, 1.30) | 0.11 | 1.35 (1.22, 1.49) | 0.41 |
|  | Never smoking | 1.25 (1.11, 1.41) |  | 1.36 (1.10, 1.70) |  | 1.27 (1.14, 1.41) |  |
|  |  |  |  |  |  |  |  |
|  | Not depression | 1.16 (1.07, 1.27) | 0.89 | 1.11 (0.95, 1.29) | 0.32 | 1.29 (1.19, 1.39 | 0.83 |
|  | Depression | 1.18 (0.95, 1.48) |  | 1.37 (0.93, 2.02) |  | 1.32 (1.06, 1.64) |  |
|  |  | Hypertension | P-int | Diabetes | P-int | Obesity | P-int |
| Smoking  habit | Overall | 1.05 (0.97, 1.13) |  | 0.92 (0.80, 1.06) |  | 0.97 (0.91, 1.05) |  |
|  |  |  |  |  |  |  |  |
|  | A level or above | 0.99 (0.90, 1.10) | 0.33 | 0.82 (0.67, 1.03) | 0.11 | 0.96 (0.88, 1.06) | 0.41 |
|  | O level or below | 1.04 (0.88, 1.22) |  | 1.04 (0.83, 1.29) |  | 0.91 (0.81, 1.01) |  |
|  |  |  |  |  |  |  |  |
|  | Not depression | 1.01 (0.93, 1.09) | 0.47 | 0.90 (0.78, 1.05) | 0.82 | 0.99 (0.92, 1.07) | 0.30 |
|  | Depression | 1.10 (0.88, 1.37) |  | 0.86 (0.59, 1.26) |  | 0.87 (0.72, 1.05) |  |
|  |  | Hypertension | P-int | Diabetes | P-int | Obesity | P-int |
| Depression | Overall | 1.53 (1.36, 1.71) |  | 1.48 (1.20, 1.82) |  | 1.17 (1.04, 1.31) |  |
|  |  |  |  |  |  |  |  |
|  | A level or above | 1.47 (1.24, 1.74) | 0.89 | 1.24 (0.90, 1.71) | 0.32 | 1.10 (0.93, 1.30) | 0.41 |
|  | O level or below | 1.49 (1.27, 1.76) |  | 1.54 (1.17, 2.02) |  | 1.12 (0.96, 1.32) |  |
|  |  |  |  |  |  |  |  |
|  | Ever smoking | 1.45 (1.21, 1.73) | 0.47 | 1.50 (1.11, 2.02) | 0.82 | 1.35 (1.14, 1.60) | 0.30 |
|  | Never smoking | 1.58 (1.35, 1.85) |  | 1.43 (1.07, 1.90) |  | 1.15 (0.99, 1.32) |  |
|  |  |  |  |  |  |  |  |
|  |  | BCS70 | | | | | |
| Mediators | Subgroups | Hypertension | P-int | Diabetes | P-int | Obesity | P-int |
| Educational  levels | Overall | 1.13 (1.04, 1.24) |  | 0.93 (0.76, 1.12) |  | 1.22 (1.12, 1.32) |  |
|  |  |  |  |  |  |  |  |
|  | Ever smoking | 1.09 (0.96, 1.24) | 0.91 | 0.93 (0.72, 1.20) | 0.98 | 1.27 (1.14, 1.41) | 0.61 |
|  | Never smoking | 1.08 (0.92, 1.26) |  | 0.93 (0.69, 1.27) |  | 1.21 (1.06, 1.38) |  |
|  |  |  |  |  |  |  |  |
|  | Not depression | 1.07 (0.96, 1.20) | 0.90 | 0.89 (0.71, 1.12) | 0.86 | 1.22 (1.11, 1.34) | 0.78 |
|  | Depression | 1.07 (0.86, 1.33) |  | 0.93 (0.63, 1.37) |  | 1.18 (0.98, 1.44) |  |
|  |  | Hypertension | P-int | Diabetes | P-int | Obesity | P-int |
| Smoking  habit | Overall | 1.06 (0.96, 1.16) |  | 0.96 (0.80, 1.16) |  | 0.95 (0.82, 1.17) |  |
|  |  |  |  |  |  |  |  |
|  | A level or above | 1.05 (0.93, 1.19) | 0.91 | 0.95 (0.75, 1.21) | 0.98 | 0.99 (0.75, 1.21) | 0.61 |
|  | O level or below | 1.04 (0.88, 1.22) |  | 0.96 (0.69, 1.32) |  | 0.95 (0.69, 1.42) |  |
|  |  |  |  |  |  |  |  |
|  | Not depression | 0.97 (0.87, 1.09) | 0.12 | 0.84 (0.67, 1.05) | 0.22 | 0.94 (0.67, 1.15) | 0.82 |
|  | Depression | 1.18 (0.95, 1.45) |  | 1.01 (0.75, 1.60) |  | 1.01 (0.75, 1.60) |  |
|  |  | Hypertension | P-int | Diabetes | P-int | Obesity | P-int |
| Depression | Overall | 1.51 (1.34, 1.70) |  | 1.67 (1.34, 2.07) |  | 1.17 (1.05, 1.30) |  |
|  |  |  |  |  |  |  |  |
|  | A level or above | 1.50 (1.29, 1.75) | 0.90 | 1.61 (1.23, 2.10) | 0.86 | 1.17 (1.02, 1.33) | 0.78 |
|  | O level or below | 1.51 (1.25, 1.82) |  | 1.67 (1.17, 2.40) |  | 1.13 (0.96, 1.34) |  |
|  |  |  |  |  |  |  |  |
|  | Ever smoking | 1.38 (1.17, 1.63) | 0.12 | 1.45 (1.08, 1.96) | 0.22 | 1.18 (1.02, 1.36) | 0.82 |
|  | Never smoking | 1.67 (1.40, 1.98) |  | 1.91 (1.39, 2.63) |  | 1.21 (1.04, 1.41) |  |
| Abbreviations: NCDS, National Childhood Development Study; BCS70, 1970 British Cohort Study, P-int, P value for interaction. | | | | | | | |
